# A systematic review and meta-analysis on the association between ambient air pollution and pulmonary tuberculosis

**DOI:** 10.1038/s41598-022-15443-9

**Published:** 2022-07-04

**Authors:** Christian Akem Dimala, Benjamin Momo Kadia

**Affiliations:** 1 Health and Human Development (2HD) Research Network, Douala, Cameroon; 2 Department of Medicine, Reading Hospital, Tower Health System, West Reading, PA USA; 3 Health Education and Research Organisation (HERO) Cameroon, Buea, Cameroon; 4 Department of Clinical Sciences, Liverpool School of Tropical Medicine, Liverpool, UK

**Keywords:** Environmental sciences, Diseases, Health care, Medical research, Risk factors

## Abstract

There is inconclusive evidence on the association between ambient air pollution and pulmonary tuberculosis (PTB) incidence, tuberculosis-related hospital admission and mortality. This review aimed to assess the extent to which selected air pollutants are associated to PTB incidence, hospital admissions and mortality. This was a systematic review of studies published in English from January 1st, 1946, through May 31st, 2022, that quantitatively assessed the association between PM_2.5_, PM_10_, NO_2_, SO_2_, CO, O_3_ and the incidence of, hospital admission or death from PTB. Medline, Embase, Scopus and The Cochrane Library were searched. Extracted data from eligible studies were analysed using STATA software. Random-effect meta-analysis was used to derive pooled adjusted risk and odds ratios. A total of 24 studies (10 time-series, 5 ecologic, 5 cohort, 2 case–control, 1 case cross-over, 1 cross-sectional) mainly from Asian countries were eligible and involved a total of 437,255 tuberculosis cases. For every 10 μg/m^3^ increment in air pollutant concentration, there was a significant association between exposure to PM_2.5_ (pooled aRR = 1.12, 95% CI: 1.06–1.19, p < 0.001, N = 6); PM_10_ (pooled aRR = 1.06, 95% CI: 1.01–1.12, p = 0.022, N = 8); SO_2_ (pooled aRR = 1.08, 95% CI: 1.04–1.12, p < 0.001, N = 9); and the incidence of PTB. There was no association between exposure to CO (pooled aRR = 1.04, 95% CI: 0.98–1.11, p = 0.211, N = 4); NO_2_ (pooled aRR = 1.08, 95% CI: 0.99–1.17, p = 0.057, N = 7); O_3_ (pooled aRR = 1.00, 95% CI: 0.99–1.02, p = 0.910, N = 6) and the incidence of PTB. There was no association between the investigated air pollutants and mortality or hospital admissions due to PTB. Overall quality of evidence was graded as low (GRADE approach). Exposure to PM_2.5_, PM_10_ and SO_2_ air pollutants was found to be associated with an increased incidence of PTB, while exposure to CO, NO_2_ and O_3_ was not. There was no observed association between exposure to these air pollutants and hospital admission or mortality due to PTB. The quality of the evidence generated, however, remains low. Addressing the tuberculosis epidemic by 2030 as per the 4th Sustainable Development Goal may require a more rigorous exploration of this association.

## Introduction

Pulmonary tuberculosis (PTB), a bacterial infection of the lungs caused by *Mycobacterium tuberculosis* is one of the top 10 causes of death worldwide and the leading cause of death from a single infectious agent^1^. PTB remains a global health emergency despite the significant progress that has been made worldwide in its control over the past two and a half decades^2^. Much still needs to be done to end the tuberculosis epidemic by 2030^3^ as per the World Health Organisation’s (WHO) 4th sustainable development goal (SDG). This includes addressing important predisposing factors to tuberculosis infection such as smoking, diabetes, human immunodeficiency virus (HIV) and social determinants of health such as poverty, malnutrition, poor ventilation and over-crowding among others^1,4^. A multi-faceted and multi-sectorial approach to tuberculosis prevention, case identification, management and control of its health and social determinants is therefore required^4,5^.

Air pollution, currently on several global health agendas, has rapidly become a global problem with the increasing global urbanisation, transportation-related emissions, and increased energy consumption. Air pollution could therefore be an important factor to address on the journey to ending tuberculosis as there are growing concerns of its association to increased tuberculosis-related hospital admissions and deaths^6,7^.

There is a well-known association between different air pollutants and cardio-respiratory diseases^8–15^. However, there is still no conclusive evidence of an association between PTB and outdoor air pollution despite its well-known association to indoor pollution from activities such as smoking and biomass fuel burning^16–18^ The review by Popovic et al. indicated a possible association between PM_2.5_ and PTB outcomes (incidence, hospital admissions and mortality) and reported the contrasting findings from several earlier studies on the association between PM_10_, NO_2_, and SO_2_ and PTB^19^, but did not synthesise these findings to determine to what extent these air pollutants are associated to PTB. Also, several studies have been published on this subject after the review by Popovic et al. This review therefore had as objectives to determine if there is an association between the selected air pollutants (PM_2.5_, PM_10_, NO_2_, SO_2_, CO, O_3_) and PTB incidence, hospital admissions and mortality, and to what extent, by systematically reviewing and quantitatively combining published evidence on this topic.

## Methods

This was a systematic literature review and meta-analysis of articles published in English from January 1st, 1946, through May 31st, 2022, that quantitatively assessed the association between ambient air pollution and PTB. The study protocol for this review was registered with the international prospective register of systematic reviews (PROSPERO) with trial registration number CRD42020165888 and has been published^20^. This review was reported according to The RepOrting standards for Systematic Evidence Syntheses (ROSES) for systematic review^21^ as presented in Additional file 1.

### Deviations from the protocol

There were no deviations from the published study protocol.

### Search for articles

A comprehensive search strategy (Additional file 2) combining medical subject headings (MeSH) and free-text searches for the appropriate keywords was developed by the authors and used to search the databases: Medline, Embase, Scopus and The Cochrane Library. The keywords ‘air pollution’, ‘carbon monoxide’, ‘nitrogen dioxide’, ‘sulphur dioxide’, ‘ozone’, and ‘particulate matter’ were combined with the keywords ‘tuberculosis’, ‘incidence’, ‘mortality’, ‘hospital admission’ and their respective synonyms, using the Boolean operator ‘AND’ in the search strategy. The search was run by the principal investigator (CAD), all searches were limited to the language English and grey literature search was not conducted given the lack of relevant studies from preliminary searches of the grey literature. Search dates of interest were January 1st, 1946, through May 31st, 2022. The search language was in English, and all the database searches were done on the same day, June 5th, 2022. The search was run twice to ensure replicability of results and the same results were obtained with each search run.

### Article screening and study eligibility criteria

#### Screening process

Articles returned by the search were saved on Zotero Version 5.0 reference management software and duplicates of the studies were manually removed by the principal investigator (CAD) with the assistance of the reference management software. More articles were added to the search output by the principal investigator by reviewing the reference list of relevant articles. The titles and abstracts of all the remaining articles were then independently screened for eligibility according to the set eligibility criteria by each of the two independent reviewers (CAD and BMK). The full texts of all the articles retained after the title and abstract screen, were then independently reviewed by the same two independent reviewers (CAD and BMK) for eligibility and inclusion to the analysis. The two independent reviewers compared their findings at the end of both the title and abstract screening and the full text review stages of the article selection process to ensure concordance in their final selection. There were no reviewer disagreements at all stages of the study selection process and no third reviewer to settle discordances as had been planned in the study protocol, was therefore needed due to concordance in the findings of the two independent reviewers.

#### Eligibility criteria

The following criteria were used during the article selection process to determine the eligible studies.

The following studies were included:

1. Population: Studies focused on adults aged 18 and above with PTB
2. Exposure: Studies that reported direct measurements on any of the air pollutants; carbon monoxide (CO), nitrogen dioxide (NO_2_), sulphur dioxide (SO_2_), ozone (O_3_), particulate matter ≤ 2.5 µm (PM_2.5_) and/or particulate matter ≤ 10 µm (PM_10_) in any country, region, city or locality;
3. Outcomes: Studies that reported measures of association on the risk of PTB incidence, hospital admission and/or mortality from PTB;
4. Study design/Other: Cross-sectional, case–control, cohorts, case-crossover, ecological and time-series studies that reported on the association between ambient air pollution and PTB.

The following studies were excluded:

1. Population: Studies that reported on respiratory diseases other than PTB
2. Exposure: Studies that reported on other forms of air pollution such as indoor air pollution
3. Outcomes: Studies that reported outcomes related to PTB in combination with other respiratory diseases. Studies that reported on measures of effect/association other than risk ratios and odds ratios or that provided data from which these measures could not be calculated.
4. Other: Conference abstracts, editorials, letters, opinion papers, unpublished studies, same studies published in different journals with the same or a different title.

#### Study validity assessment

Assessment of study quality of each included study was done by both independent reviewers (CAD and BMK) using the respective Study Quality Assessment Tools of the National Health Institute/National Heart, Lung and Blood Institute (NHI/NHLBI)^22^ depending on their study designs. There was no discordance in the overall rating of the quality of the eligible studies. Study quality indicators were included in the meta-regression.

The overall quality of the evidence provided by the studies with regards to the primary outcome of interest was assessed and graded as very low, low, moderate or high, using the Grading of Recommendations Assessment, Development and Evaluation (GRADE)^23^.

#### Data coding and extraction strategy

Data on the publication details, study methods and outcomes of interest were extracted from the eligible studies into a Microsoft excel office 365 data extraction sheet (Additional file 3) by the principal investigator (CAD) and independently rechecked by a second reviewer (BMK) for accuracy. The following data were extracted: First author, year of publication, study location, study design, socio-demographic and clinical characteristics of study participants, study duration, number of tuberculosis cases and new tuberculosis cases, annual incidences of tuberculosis, mean and median concentration data on air pollutants of interest (CO, NO_2_, SO_2_, O_3_, PM_2.5_ and PM_10_), data on incidence, hospital admission and mortality from tuberculosis, including measures of effect/association (risk ratios, odds ratios and percentage change in the incidence of PTB) and their respective confidence intervals, and confounders reported by the respective studies and if studies adjusted for confounders or not. PM_2.5_ and PM_10_ air pollutants were measured in µg/m^3^ and NO_2_, SO_2_ and O_3_ in parts per billion (ppb) and CO in parts per million (ppm). For studies that reported air pollutant concentrations in units other than the above, the Air Pollution Information System^24^, was used to convert air pollutant concentrations to appropriate units, taking into consideration the average yearly temperatures reported for the various cities or countries. The average annual outdoor temperature obtained from public sources was used for studies that did not report them. In studies where several measures of effect were reported for different quintiles or levels of exposure to air pollutants, the largest numerical estimates of the measures of effect were considered, to quantify the maximum extent of the association of air pollutants to PTB. When protective effects were observed among the measures of effect, the lowest numerical measures of effect were used. Default measures of effect reported by the studies were considered. Adjusted measures of effect were chosen over crude measures, and both single-pollutant models and multi-pollutant models were reported as appropriate. All data was transferred to STATA version 14.0 statistical software for analysis.

#### Potential effect modifiers/reasons for heterogeneity

Between-study heterogeneity was anticipated given the differences in study designs, settings, duration, sample sizes, and population characteristics based on review of existing literature.

#### Data synthesis and presentation

Meta-analyses were done through random effects models to account for the possibility of between-study heterogeneity. Risk ratios and odds ratios on the incidence of PTB following exposure to the selected air pollutants, and their respective confidence intervals from the various studies, were log-transformed, and the corresponding standard errors derived. Pooled summary estimates for the respective log-transformed measures of association were computed and presented on forest plots. Studies were pooled according to their study designs with ecologic studies and studies that used time-series analysis pooled together, separate from cohort and case–control studies. Heterogeneity between studies was assessed using the Cochrane’s Q test, and the I^2^ test statistic was reported as a measure of the extent of this heterogeneity. The Begg’s and Egger’s statistical tests were used for the statistical assessments of publication bias and small study effect^25,26^. All statistical tests and plots were done on STATA version 14.0 statistical software.

### Ethics approval and consent to participate

This systematic review does not require ethical approval as it entails a synthesis of data collected from several primary studies. No primary data collection from patients will be done for this systematic review.

## Results

### Review descriptive statistics

**Figure 1:**
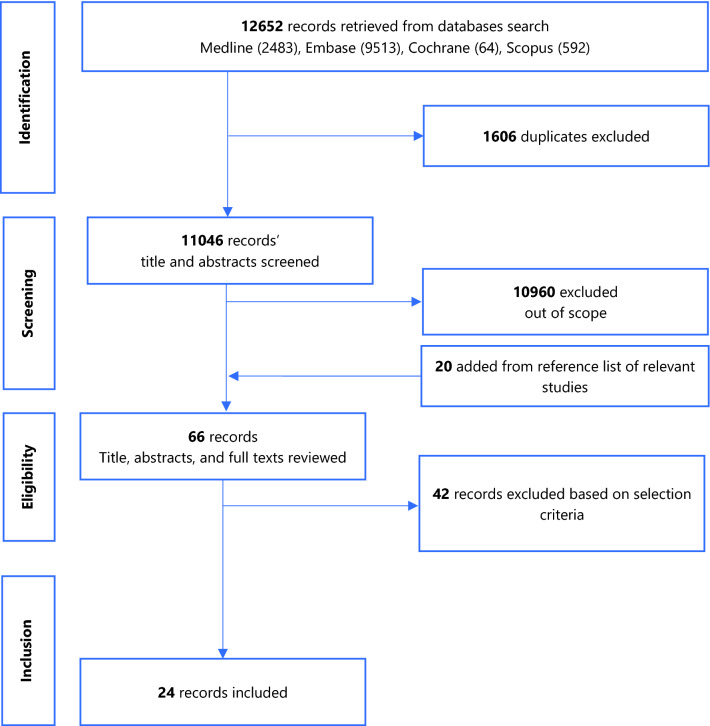
PRISMA flow chart.

Figure 1 summarises the study selection process.

A total of 12,652 records were returned by the search. Following removal of duplicates, screening of titles and abstracts, addition of studies from the reference list of relevant studies, full-text reviews, 24 eligible studies were retained. Figure 1 summarises the PRISMA flow chart of the study selection process. The studies excluded following full-text review and the reasons for exclusion are presented in Additional file 4.

### Narrative synthesis including study validity assessment

Most studies were from Asian countries and a total of 437,255 tuberculosis cases were reported across the 22 studies that reported the number of tuberculosis cases over their study periods (1996–2019)^7,27–46^. Of the 24 studies included in the review, 10 were time series, 5 were cohort studies (3 retrospective, 2 prospective), 5 were ecologic, 2 were case–control studies (1 nested, 1 retrospective), 1 was a retrospective case cross-over and 1 was cross-sectional. Average male participation was at 64.9% (N = 13 studies)^7,27,29–35,37,40,43,46^, mean age of 46.3 years (N = 7 studies)^27,30–32,37,40,43^ and average annual tuberculosis incidence was 45.3 per 100,000 population (N = 10 studies)^7,27,29,32,35,36,45–48^. Study and participant characteristics are summarised on Table 1.

**Table 1:** Study and participant characteristics of the eligible studies (N = 22 records).

Author (year)	Country (city/province)	Study design/analysis	Duration	Total TB cases	Males (%)	Mean age (years)	Annual TB incidence per 100,000
Jassal (2013)	USA (Los Angeles)	Retrospective cohort	2007–2008	111	NR	NR	NR
Hwang (2014)	South Korea (Seoul)	Retrospective cohort	2002–2006	41,185	24,952 (60.6%)	43.3	39.45
Smith (2014)	USA (North Carolina)	Ecologic	1993–2007	5319	3649 (68.6%)	NR	4.41
Alvaro-Meca (2016)	Spain (NR)	Retrospective case cross-over	1997–2012	45,427*	4577 (80.1%)	37.96	NR
Chen (2016)	Taiwan (New Taipei City)	Retrospective case–control	2010–2012	245	175 (71.4%)	59	NR
Lai (2016)	Taiwan (New Taipei City)	Prospective cohort	2005–2012	418**	37,401 (35.1%)	50.85	61
Peng (2016)	China (Shanghai City)	Prospective cohort	2003–2013	4444	3290 (74%)	NR	NR
Smith (2016)	USA (California)	Nested case–control	1996–2010	2309***	1144 (49.5%)	NR	NR
You 1 (2016)	China (Beijing)	Ecologic	2012–2014	1605	NR	NR	NR
You 2 (2016)	China (Hong-Kong)	Ecologic	2012–2015	1594	NR	NR	NR
Liu (2018)	China (Jinan)	Time series	2011–2015	9344	6230 (66.7%)	45.6	NR
Zhu (2018)	China (Chengdu)	Time series	2010–2015	36,108	24,149 (66.9%)	NR	44.15
Joob (2019)	Thailand (Bangkok)	Cross-sectional	2019–2019	0	–	NR	0
Li (2019)	China (Lianyungang)	Time-series	2014–2017	7281	5420 (74.4%)	NR	34.4
Sohn (2019)	South Korea (Seoul)	Ecologic	2009–2012	NR	NR	NR	129.6
Yao (2019)	China (Jinan)	Retrospective cohort	2014–2015	752	504 (67%)	43.7	NR
Wang (2019)	China (Shanghai)	Time-series	2013–2017	NR	NR	NR	NR
Carrasco-Escobar (2020)	Peru (Lima)	Ecologic	2015–2017	28,381	NR	NR	NR
Huang (2020)	China (Hubei)	Time-series	2015–2016	12,648	NR	NR	NR
Kim (2020)	Korea (Multiple)****	Cross-sectional time-series	2010–2016	120,280	NR	NR	NR
Liu (2020)	China (Hubei)	Ecologic	2006–2015	NR	NR	NR	91.83
Wang (2020)	China (Shijiazhuang)	Time-series	2014–2018	21,205	14,261 (67.3%)	44	NR
Yang (2020)	China (Wulumuqi)	Time-series	2013–2017	10,238	NR	NR	NR
Liu (2021)	China (Shandong)	Time-series	2013–2017	86,615	NR	NR	48.5
Xiong (2021)	China (Shanghai)	Time-series	2014–2019	1746	1076 (61.6%)	NR	0.003

*NR* not reported, *USA* United States of America, 45,427*—only 5712 with concurrent HIV were included in the study, 418**—total participants were 106,678, 2309***—total subjects were 6913, ****Multiple—Seoul, Busan, Daegu, Incheon, Gwangju, Daejeon, and Ulsan.

The average of the annual mean concentrations of the various air pollutants are presented on Table 2.

**Table 2:** Average of the annual mean and median concentrations of the air pollutants.

Air pollutant	Studies	Average of annual median concentration (min–max)	Studies	Average of annual mean concentration (min–max)
PM_2.5_ (µg/m^3^)	7	46.33 (9.6–86)	13	56.6 (15.6–100)
PM_10_ (µg/m^3^)	7	81 (20.6–154)	12	87.9 (47.7–173)
CO (ppm)	5	0.72 (0.08–11.1)	10	0.69 (0.001–1.25)
NO_2_ (ppb)	7	21.5 (11.9–34.8)	13	23.8 (13–34.4)
SO_2_ (ppb)	6	9.4 (1.2–21.4)	12	11.0 (3.2–28.4)
O_3_ (ppb)	7	42.7 (15.8–111)	11	45.1 (16–114)

*ppb* parts per billion, *ppm* parts per million.

Twelve studies were of good quality, eleven of fair quality and one of poor quality (Additional file 6). The overall quality of evidence for the association of all 6 air pollutants to the incidence of PTB was graded as low based on the study limitations affecting generalisability of the findings, and some inconsistency across the studies due to the significantly elevated between-study heterogeneity (Additional file 7).

### Data synthesis

#### Association between air pollutants and pulmonary tuberculosis Incidence

##### PM_2.5_

There was a significant association between exposure to PM_2.5_ and incidence of pulmonary tuberculosis (PTB), pooled adjusted RR = 1.12 (95% CI: 1.06–1.19), p < 0.001, N = 6, I^2^ = 72.4%^7,29,38,39,43,49^. There was no evidence of publication bias (Begg’s test, p = 0.133 and Egger’s test, p = 0.203). Begg’s test, p = 1. Likewise, Xiong et al.^46^ reported an association (RR = 3.10, 95% CI: 1.10–8.79) for a 50 µg/m^3^ increase in PM_2.5_ concentration. The study by Lai et al.^32^ (RR = 1.39, 95% CI: 0.95–2.03) which was cohort in design did not find a significant association. Jassal et al.^28^ reported an odds ratio of 25.3 (95% CI: 3.38–29.1).

##### PM_10_

**Figure 2:**
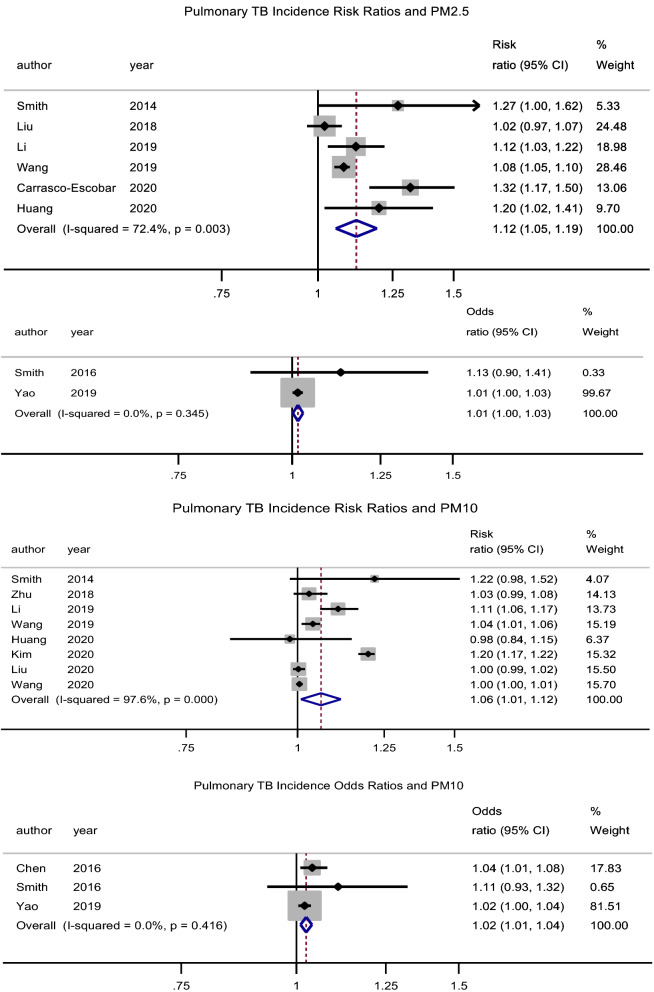
Forest plot showing the individual and pooled risk ratios and odds ratios for pulmonary tuberculosis incidence for PM_2.5_ and PM_10_. The dashed line on the Forest plot represents the overall pooled estimate. The grey squares and horizontal lines represent the vaccine acceptance rate of each study and their 95% confidence intervals. The size of the grey square represents the weight contributed by each study in the meta-analysis. The diamond represents the pooled vaccine acceptance rate and its 95% confidence intervals.

There was a significant association between exposure to PM_10_ and incidence of PTB, pooled adjusted RR = 1.06 (95% CI: 1.01–1.12), p = 0.022, N = 8, I^2^ = 97.6% (Begg’s test, p = 0.536 and Egger’s test, p = 0.204)^7,29,35,39,40,43,44,49^. The studies by Lai et al.^32^ (HR = 0.95, 95% CI: 0.78–1.17) and Hwang et al.^27^ (male RR = 1.00, 95% CI: 0.96–1.05 and female RR = 1.01, 95% CI: 0.98–1.05) did not find a significant association. Likewise, the pooled adjusted OR was 1.03 (95% CI: 1.01–1.04), p = 0.001, N = 3, I^2^ = 0% (Begg’s test, p = 1 and Egger’s test, p = 0.211) (Fig. 2)^31,34,37^.

##### CO

**Figure 3:**
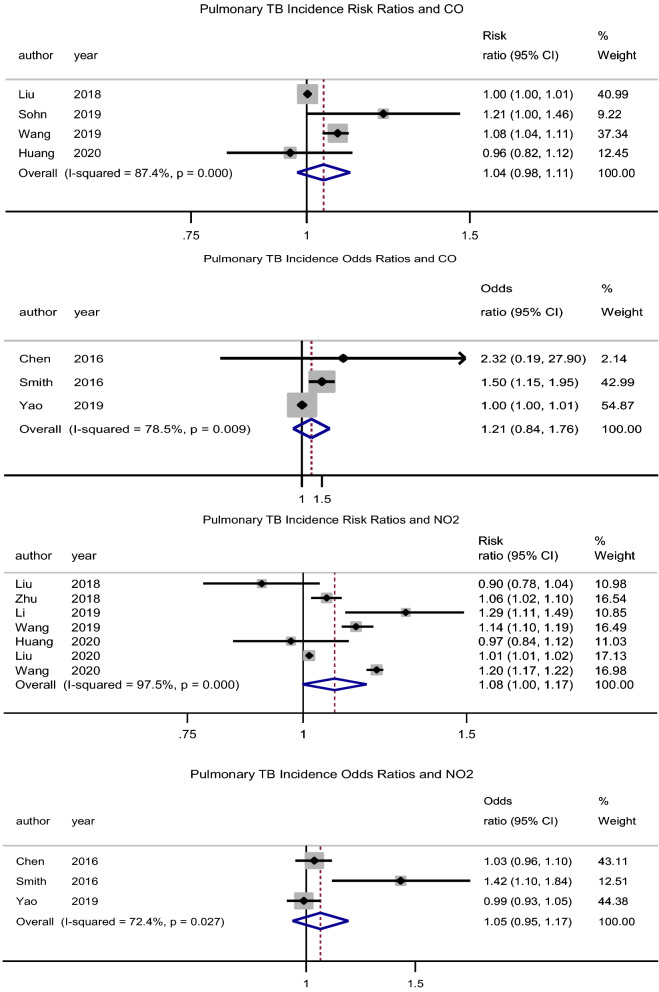
Forest plot showing the individual and pooled risk ratios and odds ratios for pulmonary tuberculosis incidence for CO and NO_2_. The dashed line on the Forest plot represents the overall pooled estimate. The grey squares and horizontal lines represent the odds ratios of each study and their 95% confidence intervals. The size of the grey square represents the weight contributed by each study in the meta-analysis. The diamond represents the pooled odds ratio and its 95% confidence intervals.

There was no significant association between exposure to CO and the incidence of PTB, pooled adjusted RR = 1.04 (95% CI: 0.98–1.11), p = 0.211, N = 4, I^2^ = 87.4% (Begg’s test, p = 0.734 and Egger’s test, p = 0.355)^39,43,47,49^. The studies by Lai et al.^32^ (HR = 1.89, 95% CI: 0.78–4.58) and Hwang et al.^27^ (male RR = 0.99, 95% CI: 0.95–1.03 and female RR = 1.01, 95% CI: 0.98–1.04) had similar findings. The pooled adjusted OR was 1.22 (95% CI: 0.84–1.76), p = 0.305, N = 3, I^2^ = 78.5% (Begg’s test, p = 1 and Egger’s test, p = 0.364) (Fig. 3)^31,34,37^. However, Xiong et al.^46^ (RR = 1.436, 95% CI: 1.004–2.053) reported a significant association for a 100 µg/m^3^ increase in CO concentration.

##### NO_2_

There was no association between exposure to NO_2_ and the incidence of PTB, pooled adjusted RR = 1.08 (95% CI: 0.99–1.17), p = 0.057, N = 7, I^2^ = 98% (Begg’s test, p = 1 and Egger’s test, p = 0.437) (Fig. 3)^7,35,39,40,43,48,49^. Lai et al.^32^ (HR = 1.33, 95% CI: 1.04–1.70) found a significant association, while Hwang et al.^27^ (male RR = 1.00, 95% CI: 0.96–1.05 and female RR = 1.01, 95% CI: 0.98–1.05) did not. Likewise, the pooled adjusted OR was 1.05 (95% CI: 0.95–1.17), p = 0.322, N = 3, I^2^ = 72.4% (Begg’s test, p = 0.296 and Egger’s test, p = 0.145) (Fig. 3)^31,34,37^. However, Xiong et al.^46^ (RR = 1.8, 95% CI: 1.11–2.91) reported a significant association for a 5 µg/m^3^ increase in NO_2_ concentration.

##### SO_2_

**Figure 4:**
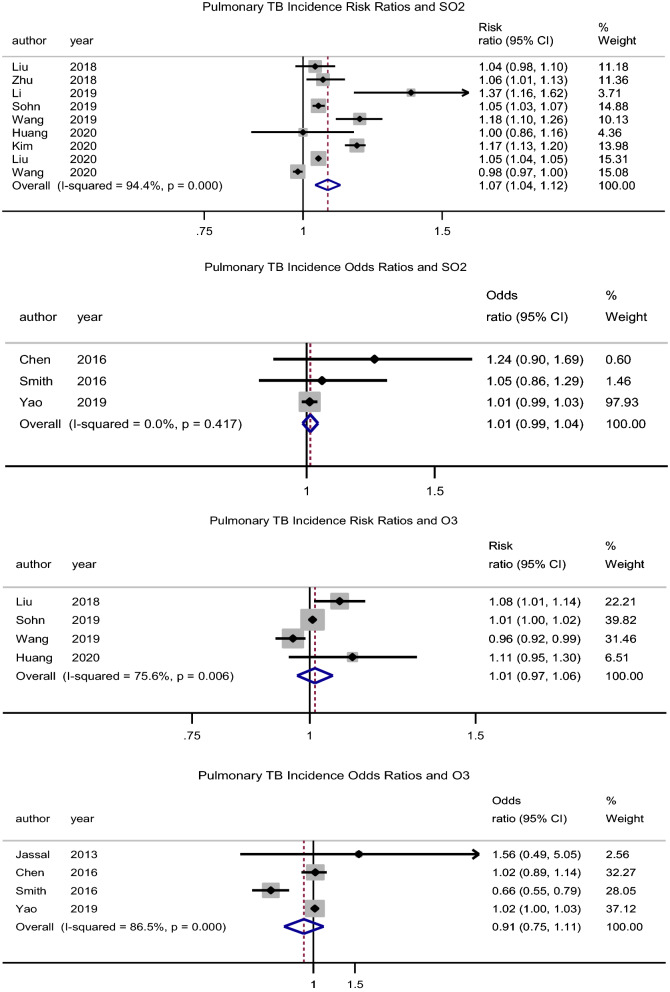
Forest plot showing the individual and pooled risk ratios and odds ratios for pulmonary tuberculosis incidence for SO_2_ and O_3_. The dashed line on the Forest plot represents the overall pooled estimate. The grey squares and horizontal lines represent the odds ratios of each study and their 95% confidence intervals. The size of the grey square represents the weight contributed by each study in the meta-analysis. The diamond represents the pooled odds ratio and its 95% confidence intervals.

There was an association between exposure to SO_2_ and incidence of PTB, pooled adjusted RR = 1.08 (95% CI: 1.04–1.12), p < 0.001, N = 9, I^2^ = 94.4% (Begg’s test, p = 0.517 and Egger’s test, p = 0.356) (Fig. 4)^7,35,39,40,43,44,47–49^. Hwang et al.^27^ (male RR = 1.07, 95% CI: 1.03–1.12 and female RR = 1.02, 95% CI: 0.98–1.07) reported similar findings in males. Likewise, Xiong et al.^46^ reported an association (RR = 1.62, 95% CI: 1.12–2.33) for a 5 µg/m^3^ increase in SO_2_ concentration.

##### O_3_

There was no significant association between O_3_ exposure and incidence of PTB, pooled adjusted RR = 1.01 (95% CI: 0.97–1.06), p = 0.560, N = 4, I^2^ = 75.6% (Begg’s test, p = 0.734 and Egger’s test, p = 0.734) (Fig. 4)^39,43,47,49^. While Hwang et al.^27^ had similar findings (male RR = 0.99, 95% CI: 0.94–1.03 and female RR = 1.01, 95% CI: 0.97–1.05), Lai et al.^32^ rather found a protective effect (HR = 0.69, 95% CI: 0.49–0.98). Xiong et al.^46^ reported an association (RR = 0.96, 95% CI: 0.93–1.0) for a 5 µg/m^3^ increase in O_3_ concentration.

Table 3 summarises the percentage change in the number of PTB cases for the respective changes in air pollutant concentrations.

**Table 3:** Percentage change in the number of pulmonary tuberculosis cases with changes in air pollutant concentrations.

Author (year)	Change in air pollutant concentration	Change in number of PTB cases (PTB incidence)
**PM**_**2.5**_		
You 1 (2016)	10 µg/m^3^ increment	3% (1.79–4.70)
You 2 (2016)	10 µg/m^3^ increment	3% (1.56–5.18)
Joob (2019)	-	0%
Wang (2019)	10 µg/m^3^ increment	8%
Yang (2020)	1 mg/m^3^	0.09%
Liu (2021)	1 µg/m^3^ increment	3.04% (2.98–3.11)
**PM**_**10**_		
Chen (2016)	1 µg/m^3^ increment	4%
Wang (2019)	10 µg/m^3^ increment	4%
Yang (2020)	1 mg/m^3^	0.08%
**CO**		
Wang (2019)	0.1 mg/m^3^ increment	8%
Yang (2020)	1 mg/m^3^	6.9%
Liu (2021)	1 µg/m^3^ increment	0.007% (0.003–0.01)
**NO**_**2**_		
Wang (2019)	10 µg/m^3^ increment	14%
Yang (2020)	1 mg/m^3^	0.42%
Liu (2021)	1 µg/m^3^ increment	1.58% (1.54–1.62)
**SO**_**2**_		
Wang (2019)	10 µg/m^3^ increment	18%
Yang (2020)	1 mg/m^3^	0.58%
Liu (2021)	1 µg/m^3^ increment	1.33% (1.29–1.37)
**O**_**3**_		
Wang (2019)	10 µg/m^3^ increment	− 4%
Yang (2020)	1 mg/m^3^	0.57%
Liu (2021)	1 µg/m^3^ increment	0.72% (0.68–0.75)

*PM*_*2.5*_ particulate matter 2.5, *PM*_*10*_ particulate matter 10, *CO* carbon monoxide, *NO*_*2*_ nitric oxide, *SO*_*2*_ sulphur dioxide, *O*_*3*_ ozone.

#### Association between air pollutants and hospital admissions and mortality due to pulmonary tuberculosis

Two studies reported a significant association between PM_2.5_ and PTB mortality; OR = 1.46 (95% CI: 1.15–1.85)^33^ and percentage change in cases of 0.08% (95% CI: 0.06–0.09)^45^. There was no significant association between CO, SO_2_, and O_3_ and PTB mortality^47^ (Table 4). Likewise, there was no significant association between PM_10_, CO, SO_2_, O_3_ and hospital admission^30,47^. NO_2_ was associated with hospital admission due to PTB, OR: 1.21 (95% CI: 1.10–1.33) (Table 4).

**Table 4:** Association between air pollutants and hospital admissions and mortality due to pulmonary tuberculosis.

Study	Air pollutant	Air pollutant concentration increment	Measure of association
**Mortality**			
Peng (2016)	PM_2.5_	2.06 µg/m^3^	OR: 1.46 (95% CI: 1.15–1.85)
Sohn (2019)	CO	1 ppb	RR: 1.70 (95% CI: 0.67–4.31)
Sohn (2019)	SO_2_	1 ppb	RR: 1.06 (95% CI: 0.99–1.13)
Sohn (2019)	O_3_	1 ppb	RR: 0.98 (95% CI: 0.94–1.01)
Liu (2021)	PM_2.5_	1 µg/m^3^	% Increase: 0.08% (0.06–0.09)
Liu (2021)	CO	1 µg/m^3^	% Increase 0.003% (0.001–0.0004)
Liu (2021)	SO_2_	1 µg/m^3^	% Increase: 0.12% (0.11–0.14)
Liu (2021)	O_3_	1 µg/m^3^	% Increase: 0.38% (0.34–0.41)
Liu (2021)	NO_2_	1 µg/m^3^	% Increase: 0.07% (0.03–0.11)
**Hospital admission**			
Alvaro-Meca (2016)	PM_10_	NM	OR: 0.97 (95% CI: 0.90–1.06)
Alvaro-Meca (2016)	CO	NM	OR: 0.92 (95% CI: 0.85–1.00)
Alvaro-Meca (2016)	NO_2_	NM	OR: 1.21 (95% CI: 1.10–1.33)
Alvaro-Meca (2016)	SO_2_	NM	OR: 0.92 (95% CI: 0.86–0.99)
Alvaro-Meca (2016)	O_3_	NM	OR: 1.03 (95% CI: 0.93–1.14)
Sohn (2019)	CO	1 ppb	OR: 1.70 (95% CI: 0.67–4.31)

*PM*_*2.5*_ particulate matter 2.5, *PM*_*10*_ particulate matter 10, *CO* carbon monoxide, *NO*_*2*_ nitric oxide, *SO*_*2*_ sulphur dioxide, *O*_*3*_ ozone, *ppb* parts per billion, *NM* not mentioned.

#### Subgroup analysis and meta-regression

Studies were categorised according to their duration (less than 5 years and 5 years or more), location (Asia and others), number of PTB cases (less than 5000 and 5000 or more) and study quality (good and fair/poor). None of these study characteristics could explain the observed heterogeneity across studies, except for study location with regards to exposure to PM_2.5_ air pollutant. There was a higher risk of PTB incidence with PM_2.5_ exposure in studies conducted out of Asia (Additional file 5).

## Discussion

Exposure to PM_2.5_, PM_10_ and SO_2_ air pollutants was found to be associated with an increased incidence of PTB, while exposure to CO, NO_2_ and O_3_ was not. There was no observed association between exposure to these air pollutants and hospital admission or mortality due to PTB. The findings of this review are particularly relevant given the increasing global concentrations and exposure to some air pollutants such as SO_2_ and PM_2.5_ over the past decades^50,51^. Public health strategies aimed at ending the tuberculosis epidemic would therefore have to work alongside interventions aimed at improving overall air quality and addressing air pollution^51^.

Air pollutants including O_3_ and NO_2_ mainly originate from volatile organic compounds, combustion processes including heating, power generation, the engines of vehicles and ships and also from industry emissions^52^. SO_2_ originates from the burning of fossil fuels for power generation and the smelting of sulfur-containing mineral ores^52^. PM_2.5_ and PM_10_ which consist of particles of organic and inorganic substances are typically suspended in the air^52^. Air pollutants have been previously associated with the development of cardio-respiratory diseases in both children and adults^8,9,11^. Traffic-related pollution and several air pollutants such as O_3_, NO_2_, PM_2.5_ and PM_10_, have not only been associated with exacerbations of asthma and chronic obstructive pulmonary disease, but have also been implicated in the development of these conditions especially in childhood^11,53,54^. Air pollutants are known to increase the risk of infection when inhaled as they dampen the natural defence barriers of the respiratory tract, inhibit muco-ciliary clearance, inhibit macrophages and initiate a chronic inflammatory response with the release of pro-inflammatory mediators^55,56^. In a similar way, exposure to particulate matter for example has immunomodulatory effects on antimycobacterial activity through impaired expression of important cytokines and chemokines which are important in controlling mycobacterial infections^57,58^. This reduced antimycobacterial host immune response predisposes to tuberculosis infection.

Measures and policies in various sectors such as the transport, housing and industry sectors are known to reduce air pollutions, including; prioritising walking and cycling in cities, using low-emission vehicles; using clean technologies that reduce industrial emissions; improving access to clean household energy for heating, lighting and cooking; making cities more green; using low-emission fuels and combustion-free power sources, among others^52^. In 2015, the WHO member states adopted a resolution for enhanced global response to the adverse health effects of air pollution, and the WHO has been overseeing this response through; the production of air quality guidelines and exposure limits to these air pollutants^52^.

Even though the studies by Ge et al.^59^ and Xu et al.^60^ reported a possible association between short-term exposure to SO_2_, our review did not assess outpatient PTB visits as an outcome. This is therefore a subject amenable to further exploration.

The studies in this review were conducted over a 24-year period and we did not observe a particular change or variation in the trend of the reported associations between exposure to the air pollutants and PTB incidence over time across the older and newer studies. Close to four fifth of the studies in our review were conducted in Asia and up to half of the studies were in China, which could affect the generalisability of the findings of this review, however, China is still a high-burden country for tuberculosis^61,62^. The observed between-study heterogeneity highlights the need for more uniform study designs and methods in future studies aiming to assess this association.

Interpretation of the findings from this review should take into consideration some limitations. This review did not assess the contribution of indoor air pollution and other comorbidities to the increased risk of PTB incidence, hospital admission and mortality. The different study designs and methodologies affected the types of confounders that could be adjusted for in the different studies and therefore introducing inconsistency in the adjustment of confounders across studies. This review, therefore, focused on the strongest reported associations between air pollutant exposure and PTB incidence rather than on the duration of exposure to the air pollutants.

## Conclusion

Exposure to PM_2.5_, PM_10_, NO_2_ and SO_2_ air pollutants was found to be associated with an increased incidence of PTB, while exposure to CO and O_3_ was not. These findings of this study and the overall quality of the evidence highlight the need for more rigorous exploration of this association.

## Supplementary Information

Supplementary Information 1.

Supplementary Information 2.

Supplementary Information 3.

Supplementary Information 4.

Supplementary Information 5.

Supplementary Information 6.

Supplementary Information 7.

Supplementary Information 8.

## Data Availability

The datasets used and/or analysed during the current study available from the corresponding author on reasonable request.
