# Supplementary material for: A systematic review and meta-analysis on the association between ambient air pollution and pulmonary tuberculosis
Source: Sci Rep. 2022 Jul 4;12:11282. doi: 10.1038/s41598-022-15443-9 (PMC9253106; doi:10.1038/s41598-022-15443-9)
Supplement: Supplementary file 2 — Supplementary Information 2. [file 41598_2022_15443_MOESM2_ESM.pdf]

Search Strategy for study on the association between ambient air pollution and pulmonary tuberculosis

Ovid Medline and Embase

Search #	Search words
1	Air pollution (MeSH terms) OR air pollution OR ambient air pollution OR outdoor air pollution OR air pollutants OR environmental pollutants OR Carbon monoxide OR CO OR Nitrogen dioxide OR NO_2 OR Sulphur dioxide OR SO_2 OR Ozone OR O_3 OR Particulate matter OR PM_{10}
2	Tuberculosis (MeSH) OR tuberculosis OR pulmonary tuberculosis OR TB OR mycobacterial infection OR mycobacterium OR drug-resistant tuberculosis OR multidrug-resistant tuberculosis OR MDR-TB OR XDR-TB
3	Incidence (MeSH) OR hospital admission (MeSH) OR hospitalisation (MeSH) OR mortality (MeSH) OR incidence OR admission OR mortality OR death
4	#1 AND #2 AND #3

MeSH – Medical Subject Headings

The Cochrane Library

Search #	Search words
1	MeSH descriptor: [Air Pollution] in all MeSH products
2	Air pollution OR ambient air pollution OR outdoor air pollution OR air pollutants OR environmental pollutants Carbon monoxide OR CO OR Nitrogen dioxide OR NO_2 OR Sulphur dioxide OR SO_2 OR Ozone OR O_3 OR Particulate matter OR PM_{10}
3	#1 OR #2
4	MeSH descriptor: [Tuberculosis] explode all trees
5	Tuberculosis OR pulmonary tuberculosis OR TB OR mycobacterial infection OR mycobacterium OR drug-resistant tuberculosis OR multidrug-resistant tuberculosis OR MDR-TB OR XDR-TB
6	#4 OR #5
7	MeSH descriptor: [Incidence] explode all trees
8	MeSH descriptor: [Mortality] explode all trees
9	Incidence OR admission OR mortality OR death
10	#7 OR #8 OR #9
11	#3 AND #6 AND #10

MeSH – Medical Subject Headings

Scopus

Search words
(air AND pollution OR ambient AND air AND pollution OR outdoor AND air AND pollution OR air AND pollutants OR environmental AND pollutants AND carbon AND monoxide OR co OR nitrogen AND dioxide OR no2 OR sulphur AND dioxide OR so2 OR ozone OR o3 OR particulate AND matter OR pm10) AND (tuberculosis OR pulmonary AND tuberculosis OR tb OR mycobacterial AND infection OR mycobacterium OR drug-resistant AND tuberculosis OR multidrug-resistant AND tuberculosis OR mdr-tb OR xdr-tb) AND (incidence OR admission OR mortality OR death)
