# Supplementary material for: A systematic review and meta-analysis on the association between ambient air pollution and pulmonary tuberculosis
Source: Sci Rep. 2022 Jul 4;12:11282. doi: 10.1038/s41598-022-15443-9 (PMC9253106; doi:10.1038/s41598-022-15443-9)
Supplement: Supplementary file 4 — Supplementary Information 4. [file 41598_2022_15443_MOESM4_ESM.pdf]

Excluded Studies and reasons for exclusion.

Author, Publication year	Reason for Exclusion
Pompilio, 2020[1]	Review study on air pollution and respiratory diseases including tuberculosis, however, only few studies on the association between air pollution and PTB are listed in the review
Baris, 2004[2]	Study out of scope and not investigating if ambient air pollution is associated to PTB
Blount, 2017[3]	Study assessed traffic volumes and traffic densities as exposures of interest rather than specific air pollutants, and their association to mortality during TB treatment
Blount, 2020[4]	Study was a conference abstract and did not provide other important variables such as selection of participants, measurement of air pollutants or mean air pollutant concentrations
Bykhovskii A.V., 1979[5]	Study out of scope and not investigating if ambient air pollution is associated to PTB
Chong, 2022[6]	Study effectively discussed the effect of SO2 and PM2.5 on TB hospitalisation, but did not provide numerical measures of association between the air pollutants and the outcome which could have enable addition of the study in the meta-analysis
Cohen, 2007[7]	Report on the possible association between PTB and air pollution but not an actual study
Feng, 2017[8]	Study discussing the health effects of ambient PM2.5 but not its association to PTB
Ge, 2017[9]	Study reports on the association of SO2 to initial outpatient PTB rather than PTB incidence, admission and mortality
Hargreaves, 2011[10]	This article reports on the social determinants of tuberculosis and does not assess the association between air pollutants and PTB
Huang, 2020[11]	The Study outcome (TB outpatient visits) was different from those of our study (TB incidence, hospital admission and mortality)
Kan, 2004[12]	Study discusses the human health and economic impact of particulate air pollution, and not the association between particulate air pollution and PTB
Katoto, 2019[13]	The study summarised existing evidence on the effect of ambient air pollution on health outcomes in sub-Saharan Africa, but not with a particular focus on PTB as a health outcome
Khalilzadeh, 2009[14]	This study assessed the association between air pollutants and asthma and cardiovascular diseases, and not PTB
Khilnani, 2018[15]	The study summarises the adverse effects of air pollution in India but does not look at its association to PTB
Koshal, 1976[16]	The paper examines TB mortality models with varying levels of air pollution, but does not specifically examine the association between specific air pollutants and PTB
Kurtulus, 2021[17]	The studies does not report appropriate measures of association between air pollutants and tuberculosis risk to enable inclusion in the synthesis and meta-analysis
Laumbach, 2012[18]	The study reports on the fact that there is still no evidence on the association between traffic-related air pollution and PTB

Excluded Studies and reasons for exclusion continued…

Author, Publication year	Reason for Exclusion
Li, 2017[19]	The study examines the health effects of indoor particulate matter rather than outdoor particulate matter. PTB was not assessed as a health outcome
Lin 2019[20]	The study reported population attributable factor for 4 different cities rather than a combined summary estimate as odds ratios or risk ratios
Matyasovszky, 2011[21]	The study assessed the role of air pollution and meteorological parameters in hospital admissions due to respiratory symptoms and not PTB
Morakinyo, 2017[22]	The study examine the adverse health effects in general rather than PTB in particular, of various air pollutants
Patella, 2018[23]	This literature review looked at the effect of air pollution on respiratory disease and not PTB in particular
Petersen, 2019[24]	This study assessed the association between air pollution and PTB. It reports on both indoor and outdoor air pollution, however, the outdoor pollution sources assessed are outdoor cooking fires and trash burning and the specific pollutants of interests are not reported
Popovic, 2019[25]	This is a systematic literature review and not primary research study
Rajaei, 2019[26]	This study studies the association between air pollution and TB risk using a GIS approach. The study, however, did not report essential measures of effect that quantified the risk of PTB with exposure to the various air pollutants
Rao, 2016[27]	Air pollution was not one of the meteorological determinants of PTB assessment in this study
Rivas, 2013[28]	The study examined the effect of particulate matter on the anti-mycobacterial innate immunity, but did not assess if this air pollutant is quantitatively associated with PTB incidence, hospital admission or mortality
Saravia, 2013[29]	This review examines the health effects on particulate matter, but does not assess for any association of this air pollutant to PTB
Schmidt, 2018[30]	This is a report exploring a few previous studies on the potential association between air pollution and PTB, it, however, is not a study on the association between air pollution and PTB
Severs, 1978[31]	This study reports on the association of air pollutants to ischaemic heart disease and not PTB
Shafiei, 2019[32]	The study looks at the effect of exposure to silica dust exposure on PTB risk, and not the effect of the air pollutants of interest in our study
Smith, 2003[33]	The study explores the disease burden due to indoor air pollution and not ambient air pollution
Subbayya, 1981[34]	The study reports on the relationship between air pollution and incidence of respiratory diseases
Tang, 2017[35]	The study reports on the mortality from circulatory and respiratory diseases in general due to air pollution
Wang 2022[36]	The study reports on the effect of ambient air pollution on the risk of tuberculosis outpatient visits and not tuberculosis incidence, hospital admission or mortality
WHO, 1995[37]	Though this study reports the risk of TB associated with air pollution, the pollution is stratified according to levels of pollution (low, medium and high pollution areas) instead of particular pollutants, Implying the pollutant effect of SO_2 and total suspended particulate was combined to produce an overall effect.

Excluded Studies and reasons for exclusion continued…

Author, Publication year	Reason for Exclusion
Wong, 2017[38]	This study reports on the association between PM2.5 and PTB, however this is an abstract and the data retrieved from the abstract is inadequate
Xia, 2017[39]	This study reports on the effects of ambient air PM 25 exposure on human respiratory system
Xu, 2019[40]	The study outcome (Initial TB outpatient visits) was different from those of our study (TB incidence, hospital admission and mortality)
Yasri, 2021[41]	The study reports on the correlation between air SO_2 concentration and tuberculosis incidence, but does not report on other measures of association of interest as per the study inclusion criteria such as risk ratios, odds ratios or percentage changes
You, 2016[42]	This study reports on the association of PM2.5 to PTB, however, the study compares the case of Hong Kong and Beijing. No overall/combined appropriate measures of effect could be calculated from the presented data

References

1. Pompilio A, Bonaventura GD. Ambient air pollution and respiratory bacterial infections, a troubling association: epidemiology, underlying mechanisms, and future challenges. Crit Rev Microbiol. 2020 Sep 2;46(5):600–30.

2. Baris E, Ezzati M. Should interventions to reduce respirable pollutants be linked to tuberculosis control programmes?. BMJ. 2004;329(7474):1090–3.

3. Blount Robert J., Pascopella Lisa, Catanzaro Donald G., Barry Pennan M., English Paul B., Segal Mark R., et al. Traffic-Related Air Pollution and All-Cause Mortality during Tuberculosis Treatment in California. Environ Health Perspect. 125(9):097026.

4. Blount R j., Phan H, Trinh T, McLaughlin R, Huy Han D, Zabner J, et al. Urban Air Pollution and Tuberculosis Infection in Vietnam: Findings from an Ongoing Prospective Household Contacts Study. In: D26 CLINICAL AND EPIDEMIOLOGICAL DEVELOPMENTS IN TB [Internet]. American Thoracic Society; 2020 [cited 2021 Jan 28]. p. A6380–A6380. (American Thoracic Society International Conference Abstracts). Available from: https://www.atsjournals.org/doi/abs/10.1164/ajrccm-conference.2020.201.1_MeetingAbstracts.A6380

5. Bykhovskii A.V., Diubankova E.N. Several findings concerning the effect of atmospheric pollution on the health of a population. Gig Sanit. 1979;(6):51–5.

6. Chong KC, Yeoh EK, Leung CC, Lau SYF, Lam HCY, Goggins WB, et al. Independent effect of weather, air pollutants, and seasonal influenza on risk of tuberculosis hospitalization: An analysis of 22-year hospital admission data. Sci Total Environ. 2022 May 4;837:155711. doi: 10.1016/j.scitotenv.2022.155711. Epub ahead of print. PMID: 35523336.

7. Cohen A., Mehta S. Pollution and tuberculosis: Outdoor sources [9]. PLoS Med. 2007;4(3):600.

8. Feng S, Gao D, Liao F, Zhou F, Wang X. The health effects of ambient PM2.5 and potential mechanisms. Ecotoxicol Environ Saf. 2016;128:67–74.

9. Ge E, Fan M, Qiu H, Hu H, Tian L, Wang X, et al. Ambient sulfur dioxide levels associated with reduced risk of initial outpatient visits for tuberculosis: A population-based time series analysis. Environ Pollut. 2017;228:408–15.

10. Hargreaves JR, Boccia D, Evans CA, Adato M, Petticrew M, Porter JDH. The Social Determinants of Tuberculosis: From Evidence to Action. Am J Public Health. 2011 Apr 1;101(4):654–62.

11. Huang K, Ding K, Yang X-J, Hu C-Y, Jiang W, Hua X-G, et al. Association between short-term exposure to ambient air pollutants and the risk of tuberculosis outpatient visits: A time-series study in Hefei, China. Environ Res. 2020 May;184:109343.
12. Kan H, Chen B. Particulate air pollution in urban areas of Shanghai, China: Health-based economic assessment. Sci Total Environ. 2004;322(1–3):71–9.
13. Katoto PDMC, Byamungu L, Brand AS, Mokaya J, Strijdom H, Goswami N, et al. Ambient air pollution and health in Sub-Saharan Africa: Current evidence, perspectives and a call to action. Environ Res. 2019;173:174–88.
14. Khalilzadeh S., Khalilzadeh Z., Emami H., Masjedi M.R. The relation between air pollution and cardiorespiratory admissions in Tehran. Tanaffos. 2009;8(1):35– 40.
15. Khilnani GC, Tiwari P. Air pollution in India and related adverse respiratory health effects: Past, present, and future directions. Curr Opin Pulm Med. 2018;24(2):108–16.
16. Koshal RK, Koshal M. Air pollution and tuberculosis disease mortality - a quantitative analysis (A viewpoint). Int J Environ Stud. 1976 Jan 1;9(2):105–10.
17. Kurtuluş Ş, Can R. The Effect of Environmental Exposures on the Diagnosis of Tuberculosis in Syrian Refugees. Turk Thorac J. 2021 Nov;22(6):489-493. doi: 10.5152/TurkThoracJ.2021.21158. PMID: 35110266; PMCID: PMC8975346.
18. Laumbach RJ, Kipen HM. Respiratory health effects of air pollution: update on biomass smoke and traffic pollution. J Allergy Clin Immunol. 2012;129(1):3– 3.
19. Li Z, Wen Q, Zhang R. Sources, health effects and control strategies of indoor fine particulate matter (PM2.5): A review. Sci Total Environ. 2017;586:610–22.
20. Lin Y-J, Lin H-C, Yang Y-F, Chen C-Y, Ling M-P, Chen S-C, et al. Association Between Ambient Air Pollution and Elevated Risk of Tuberculosis Development. Infect Drug Resist. 2019 Dec 6;12:3835–47.
21. Matyasovszky I, Makra L, Bálint B, Guba Z, Sümeghy Z. Multivariate analysis of respiratory problems and their connection with meteorological parameters and the main biological and chemical air pollutants. Atmos Environ. 2011;45(25):4152–9.
22. Morakinyo OM, Adebowale AS, Mokgobu MI, Mukhola MS. Health risk of inhalation exposure to sub-10 µm particulate matter and gaseous pollutants in an urban-industrial area in South Africa: An ecological study. BMJ Open [Internet]. 2017;7(3). Availablefrom: https://www.scopus.com/inward/record.uri?eid=2-s2.0-85015273140&doi=10.1136%2fbmjopen-2016- 013941&partnerID=40&md5=d6b05000cdfd0601a5c07feac97e58ed
23. Patella V, Florio G, Magliacane D, Giuliano A, Crivellaro MA, Bartolomeo D, et al. Urban air pollution and climate change: "The Decalogue: Allergy Safe Tree" for allergic and respiratory diseases care. Clin Mol Allergy. 2018;16(1):1.
24. Petersen AB, Muffley N, Somsamouth K, Singh PN. Smoked Tobacco, Air Pollution, and Tuberculosis in Lao PDR: Findings from a National Sample. Int J Environ Res Public Health. 2019 Jan;16(17):3059.
25. Popovic I, Magalhaes RJS, Ge E, Marks GB, Dong G-H, Wei X, et al. A systematic literature review and critical appraisal of epidemiological studies on outdoor air pollution and tuberculosis outcomes. Environ Res. 2019;170:33–45.
26. Rajaei E, Hadadi M, Madadi M, Aghajani J, Ahmad M, Farnia P, et al. Outdoor air pollution affects tuberculosis development based on geographical information system modeling. Biomed Biotechnol Res J. 2018 Jan 1;2(1):39–39.
27. Rao H-X, Zhang X, Zhao L, Yu J, Ren W, Zhang X-L, et al. Spatial transmission and meteorological determinants of tuberculosis incidence in Qinghai Province, China: A spatial clustering panel analysis. Infect Dis Poverty [Internet]. 2016;5(1). Available from: https://www.scopus.com/inward/record.uri?eid=2-s2.0- 85006224620&doi=10.1186%2fs40249-016-0139-4&partnerID=40&md5=6f25d83829c83d56c7e5a13fc478ec88

28. Rivas C.E., Cantarella P., Sarkar S., Rockafellow M., Osornio Vargas A.R., Torres M., et al. Particulate air pollution matter (PM) modifies innate immunity of type II pneumocytes (A549) against mycobacterium tuberculosis. Am J Respir Crit Care Med [Internet]. 2014;189(MeetingAbstracts). Available from: http://www.atsjournals.org/doi/pdf/10.1164/ajrccm-conference.2014.189.1_MeetingAbstracts.A2489
29. Saravia J, Lee GI, Lomnicki S, Dellinger B, Cormier SA. Particulate Matter Containing Environmentally Persistent Free Radicals and Adverse Infant Respiratory Health Effects: A Review. J Biochem Mol Toxicol. 2013;27(1):56–68.
30. Schmidt S. TB and tailpipes: Does traffic-related air pollution affect mortality during tuberculosis treatment? Environ Health Perspect. 2018;126(2):024005.
31. Severs R., Whitehead L., Lane R. Air quality correlates of chronic disease mortality: Harris County, Texas 1969-1971. Tex Rep Biol Med. 1978; VOL. 36((Severs, Whitehead, Lane) Sch. Publ. Hlth, Univ. Texas Hlth Sci. Cent., Houston, Tex. 77025 United States):169–84.
32. Shafiei M., Ghasemian A., Eslami M., Nojoomi F., Rajabi-Vardanjani H. Risk factors and control strategies for silicotuberculosis as an occupational disease. New Microbes New Infect. 2019;27((Shafiei) Department of Microbiology, Pasteur Institute of Iran, Tehran, Iran, Islamic Republic of):75–7.
33. Smith KR, Mehta S. The burden of disease from indoor air pollution in developing countries: comparison of estimates. Int J Hyg Environ Health. 2003;206(4– 5):279–89.
34. Venkata Subbayya N., Kotaiah B., Ramprasad G., Pratapa Mowli P. Study on the relation between respiratory diseases and air pollution with special reference to Surat city. Indian J Environ Prot. 1981;1(1):15–20.
35. Tang G., Zhao P., Gao W., Cheng M., Xin J., Li X., et al. Mortality and air pollution in Beijing: The long-term relationship. Atmos Environ. 2017;150((Tang, Wang, Gao, Cheng, Xin, Wang) State Key Laboratory of Atmospheric Boundary Layer Physics and Atmospheric Chemistry (LAPC), Institute of Atmospheric Physics, Chinese Academy of Sciences, Beijing 100029, China):238–43.
36. Wang XQ, Li YQ, Hu CY, Huang K, Ding K, Yang XJ, et al. Short-term effect of ambient air pollutant change on the risk of tuberculosis outpatient visits: a time-series study in Fuyang, China. Environ Sci Pollut Res Int. 2022 Apr;29(20):30656-30672. doi: 10.1007/s11356-021-17323-7. Epub 2022 Jan 7. PMID: 34993790.
37. World Health Organization Office of Global and Integrated Environmental. Air pollution and its health effects in China: a monograph. 1995 [cited 2020 Feb 4]; Available from: https://apps.who.int/iris/handle/10665/63561
38. Wong N.S., Leung C.C., Li Y., Poon C.M., Yao S., Wong E.L.Y., et al. PM2.5 concentration and elderly tuberculosis: Analysis of spatial and temporal associations. The Lancet. 2017;390(SPEC.ISS 1):68.
39. Xia Y, Fan L, Guan Y, Liu S, Xiao Y. Progress in research of relationship between ambient air PM 25 and lung diseases. Chin J Endem. 2017;38(7):993–6.
40. Xu M, Liao J, Yin P, Hou J, Zhou Y, Huang J, et al. Association of air pollution with the risk of initial outpatient visits for tuberculosis in Wuhan, China. Occup Environ Med. 2019 Aug 1;76(8):560–6.
41. Yasri S, Wiwanitkit V. Tuberculosis incidence in area with sulfur dioxide pollution: an observation. Med Gas Res. 2021 Apr-Jun;11(2):58-60. doi: 10.4103/2045-9912.311490. PMID: 33818444; PMCID: PMC8130663.
42. You S, Tong YW, Neoh KG, Dai Y, Wang C-H. On the association between outdoor PM2.5 concentration and the seasonality of tuberculosis for Beijing and Hong Kong. Environ Pollut Barking Essex 1987. 2016 Nov; 218:1170–9.
