# Supplementary material for: A systematic review and meta-analysis on the association between ambient air pollution and pulmonary tuberculosis
Source: Sci Rep. 2022 Jul 4;12:11282. doi: 10.1038/s41598-022-15443-9 (PMC9253106; doi:10.1038/s41598-022-15443-9)
Supplement: Supplementary file 5 — Supplementary Information 5. [file 41598_2022_15443_MOESM5_ESM.pdf]

Sub-group analyses and meta-regression for the air pollutants and Incidence of pulmonary tuberculosis

Air pollutants and study Characteristics	Strata	# of Studies	RR (95% CI)	Within-stratum P value (I^2)	Meta-regression relative RR	Between-stratum P value
**$PM_{2.5}$**						
Study duration	< 5 years	3	1.20 (1.08 - 1.33)	0.111 (54.6%)	1.06	0.198
	≥ 5 years	4	1.13 (1.06 - 1.20)	0.055 (60.6%)	1	
TB cases	< 5000	0	-	-	-	-
	≥ 5000	7	1.13 (1.06 - 1.20)	0.003 (69.7%)	-	
Study location	Asia	5	1.08 (1.03 - 1.14)	0.075 (53.0%)	0.55	**0.042**
	Other	2	1.96 (0.89 - 4.32)	0.790 (0%)	1	
Study quality	Good	3	1.15 (1.06 – 1.24)	0.373 (0%)	1.03	0.590
	Fair or Poor	4	1.12 (1.03 – 1.20)	0.001 (80.8%)	1	
**PM_{10}**						
Study duration	< 5 years	2	1.07 (0.95 - 1.20)	0.138 (54.5%)	1.03	0.774
	≥ 5 years	8	1.04 (1.02 - 1.06)	<0.001 (98.2%)	1	
TB cases	< 5000	0	-	-	-	-
	≥ 5000	10	1.05 (1.02 - 1.07)	<0.001 (97.8%)	-	
Study location	Asia	9	1.04 (1.02 - 1.07)	<0.001 (98.0%)	0.85	0.269
	Other	1	1.22 (0.98 - 1.52)	-	1	
Study quality	Good	5	1.09 (0.98 – 1.22)	<0.001 (97.9%)	1.04	0.102
	Fair or Poor	5	1.00 (0.99 – 1.02)	<0.001 (93.6%)	1	

Sub-group analyses and meta-regression for the air pollutants and Incidence of pulmonary tuberculosis continued…

Air pollutants and study characteristics	Strata	# of Studies	RR (95% CI)	Within-stratum P value (I^2)	Meta-regression relative RR	Between-stratum P value
**CO**						
Study duration	< 5 years	2	1.07 (0.85 - 1.34)	0.066 (70.4%)	1.06	0.758
	≥ 5 years	4	1.01 (1.00 - 1.02)	<0.001 (88.9%)	1	
TB cases	< 5000	0	-	-	-	-
	≥ 5000	6	1.01 (1.00 - 1.20)	<0.001 (83.9%)	-	
Study location	Asia	6	1.01 (1.00 - 1.20)	<0.001 (83.9%)	-	-
	Other	0	-	-	-	
Study quality	Good	1	1.89 [0.78 – 4.58)	-	1.87	0.295
	Fair or Poor	5	1.01 (1.00 - 1.02)	<0.001 (83.6%)	1	
**NO_2**						
Study duration	< 5 years	2	1.12 (0.85 - 1.48)	0.007 (86.4%)	1.05	0.728
	≥ 5 years	7	1.07 (1.03 - 1.12)	<0.001 (98.2%)	1	
TB cases	< 5000	0	-	-	-	-
	≥ 5000	9	1.08 (1.03 - 1.13)	<0.001 (97.7%)	-	
Study location	Asia	9	1.08 (1.03 - 1.13)	<0.001 (97.7%)	-	
	Other	0	-	-	-	
Study quality	Good	3	1.18 (0.96 – 1.45)	0.001 (86.4%)	1.12	0.319
	Fair or Poor	6	1.05 (0.96 – 1.15)	<0.001 (97.7%)	1	

Sub-group analyses and meta-regression for the air pollutants and Incidence of pulmonary tuberculosis continued…

Air pollutants and study characteristics	Strata	# of Studies	RR (95% CI)	Within-stratum P value (I^2)	Meta-regression relative RR	Between-stratum P value
**SO_2**						
Study duration	< 5 years	3	1.11 (0.96 - 1.29)	0.006 (80.6%)	1.05	0.507
	≥ 5 years	7	1.06 (1.04 - 1.09)	<0.001 (95.8%)	1	
TB cases	< 5000	0	-	-	-	-
	≥ 5000	10	1.06 (1.04 - 1.09)	<0.001 (94.1%)	-	
Study location	Asia	10	1.06 (1.04 - 1.09)	<0.001 (94.1%)	-	-
	Other	0	-	-	-	
Study quality	Good	3	1.15 (1.04 – 1.28)	<0.001 (96.6%)	1.10	0.066
	Fair or Poor	7	1.05 (1.01 – 1.08)	<0.001 (93.4%)	1	
**O_3**						
Study duration	< 5 years	2	1.02 (0.95 - 1.196)	0.224 (32.3%)	1.03	0.660
	≥ 5 years	4	0.99 (0.95 - 1.05)	0.003 (79.0%)	1	
TB cases	< 5000	0	-	-	-	-
	≥ 5000	6	1.00 (0.99 - 1.02)	0.001 (75.5%)	-	
Study location	Asia	6	1.00 (0.99 - 1.02)	0.001 (75.5%)	0.86	0.269
	Other	0	-	-	1	
Study quality	Good	1	0.69 (0.49 – 0.98)	-	0.69	0.134
	Fair or Poor	5	1.00 (0.99 – 1.02)	0.003 (75.0%)	1	
