# Supplementary material for: A systematic review and meta-analysis on the association between ambient air pollution and pulmonary tuberculosis
Source: Sci Rep. 2022 Jul 4;12:11282. doi: 10.1038/s41598-022-15443-9 (PMC9253106; doi:10.1038/s41598-022-15443-9)
Supplement: Supplementary file 6 — Supplementary Information 6. [file 41598_2022_15443_MOESM6_ESM.pdf]

Quality assessment of the included studies (for observational cohort, cross-sectional, before-after studies)

Criteria	Jassal 2013	Hwang 2014	Smith 2014	Alvaro-Meca 2014	Lai 2016	Peng 2016	You 2016	Liu 2018	Zhu 2018	Joob 2019
1. Was the research question or objective in this paper clearly stated?	Yes	Yes	Yes	Yes	Yes	Yes	Yes	Yes	Yes	Yes
2. Was the study population clearly specified and defined?	Yes	Yes	Yes	Yes	Yes	Yes	Yes	Yes	Yes	Yes
3. Was the participation rate of eligible persons at least 50%?	NR	Yes	Yes	NR	Yes	Yes	NR	Yes	Yes	NR
4. Were all the subjects selected or recruited from the same or similar populations (including the same time period)? Were inclusion and exclusion criteria for being in the study prespecified and applied uniformly to all participants?	Yes	Yes	Yes	Yes	Yes	Yes	Yes	Yes	Yes	Yes
5. Was a sample size justification, power description, or variance and effect estimates provided?	Yes	NA	NA	No	No	No	No	No	No	No
6. For the analyses in this paper, were the exposure(s) of interest measured prior to the outcome(s) being measured?	Yes	Yes	Yes	No	Yes	Yes	Yes	Yes	Yes	Yes
7. Was the timeframe sufficient so that one could reasonably expect to see an association between exposure and outcome if it existed?	CD	CD	CD	CD	CD	CD	CD	CD	CD	CD
8. For exposures that can vary in amount or level, did the study examine different levels of the exposure as related to the outcome (e.g., categories of exposure, or exposure measured as continuous variable)?	No	Yes	Yes	No	No	No	Yes	Yes	Yes	NR
9. Were the exposure measures (independent variables) clearly defined, valid, reliable, and implemented consistently across all study participants?	Yes	Yes	Yes	Yes	Yes	Yes	Yes	Yes	Yes	NR
10. Was the exposure(s) assessed more than once over time?	Yes	Yes	Yes	Yes	Yes	Yes	Yes	Yes	Yes	No
11. Were the outcome measures (dependent variables) clearly defined, valid, reliable, and implemented consistently across all study participants?	Yes	Yes	Yes	Yes	Yes	Yes	Yes	Yes	Yes	NR
12. Were the outcome assessors blinded to the exposure status of participants?	No	No	No	No	No	No	No	No	No	No
13. Was loss to follow-up after baseline 20% or less?	NA	NA	NA	NA	NA	NA	NA	NA	NA	NA
14. Were key potential confounding variables measured and adjusted statistically for their impact on the relationship between exposure(s) and outcome(s)?	Yes	No	Yes	No	Yes	Yes	No	No	No	No
**Quality rating**	**Good**	**Fair**	**Good**	**Fair**	**Good**	**Good**	**Fair**	**Fair**	**Fair**	**Poor**

CD – Cannot determine, NA – Not applicable, NR – Not reported

Quality assessment of the included studies continued…

Criteria	Li 2019	Sohn 2019	Wang 2019	Yao 2019	Carrasco-Escobar 2020	Huang 2020	Kim 2020	Liu 2020	Wang 2020	Yang 2020
1. Was the research question or objective in this paper clearly stated?	Yes	Yes	Yes	Yes	Yes	Yes	Yes	Yes	Yes	Yes
2. Was the study population clearly specified and defined?	Yes	Yes	No	Yes	Yes	Yes	Yes	Yes	Yes	Yes
3. Was the participation rate of eligible persons at least 50%?	NR	NR	NR	Yes	Yes	Yes	Yes	NR	NR	Yes
4. Were all the subjects selected or recruited from the same or similar populations (including the same time period)? Were inclusion and exclusion criteria for being in the study prespecified and applied uniformly to all participants?	Yes	Yes	NR	Yes	Yes	Yes	Yes	Yes	Yes	Yes
5. Was a sample size justification, power description, or variance and effect estimates provided?	No	No	No	No	No	No	No	No	No	NA
6. For the analyses in this paper, were the exposure(s) of interest measured prior to the outcome(s) being measured?	Yes	Yes	Yes	Yes	Yes	Yes	Yes	Yes	Yes	Yes
7. Was the timeframe sufficient so that one could reasonably expect to see an association between exposure and outcome if it existed?	CD	CD	CD	CD	CD	CD	CD	CD	CD	CD
8. For exposures that can vary in amount or level, did the study examine different levels of the exposure as related to the outcome (e.g., categories of exposure, or exposure measured as continuous variable)?	Yes	No	NA	No	No	Yes	Yes	Yes	No	No
9. Were the exposure measures (independent variables) clearly defined, valid, reliable, and implemented consistently across all study participants?	Yes	Yes	Yes	Yes	Yes	Yes	Yes	Yes	Yes	Yes
10. Was the exposure(s) assessed more than once over time?	Yes	Yes	Yes	Yes	Yes	Yes	Yes	Yes	Yes	Yes
11. Were the outcome measures (dependent variables) clearly defined, valid, reliable, and implemented consistently across all study participants?	Yes	Yes	Yes	Yes	Yes	Yes	Yes	Yes	Yes	Yes
12. Were the outcome assessors blinded to the exposure status of participants?	No	No	No	No	No	No	No	No	No	No
13. Was loss to follow-up after baseline 20% or less?	NA	NA	NA	NA	NA	NA	NA	NA	NA	NA
14. Were key potential confounding variables measured and adjusted statistically for their impact on the relationship between exposure(s) and outcome(s)?	Yes	Yes	No	Yes	No	No	Yes	Yes	No	Yes
**Quality rating**	**Good**	**Fair**	**Fair**	**Fair**	**Fair**	**Fair**	**Good**	**Good**	**Fair**	**Good**

CD – Cannot determine, NA – Not applicable, NR – Not reported

Quality assessment of the included studies continued…

Criteria	Liu 2021	Xiong 2021
1. Was the research question or objective in this paper clearly stated?	Yes	Yes
2. Was the study population clearly specified and defined?	Yes	Yes
3. Was the participation rate of eligible persons at least 50%?	Yes	Yes
4. Were all the subjects selected or recruited from the same or similar populations (including the same time period)? Were inclusion and exclusion criteria for being in the study prespecified and applied uniformly to all participants?	Yes	Yes
5. Was a sample size justification, power description, or variance and effect estimates provided?	No	NA
6. For the analyses in this paper, were the exposure(s) of interest measured prior to the outcome(s) being measured?	Yes	Yes
7. Was the timeframe sufficient so that one could reasonably expect to see an association between exposure and outcome if it existed?	CD	CD
8. For exposures that can vary in amount or level, did the study examine different levels of the exposure as related to the outcome (e.g., categories of exposure, or exposure measured as continuous variable)?	No	Yes
9. Were the exposure measures (independent variables) clearly defined, valid, reliable, and implemented consistently across all study participants?	Yes	Yes
10. Was the exposure(s) assessed more than once over time?	Yes	Yes
11. Were the outcome measures (dependent variables) clearly defined, valid, reliable, and implemented consistently across all study participants?	Yes	Yes
12. Were the outcome assessors blinded to the exposure status of participants?	No	No
13. Was loss to follow-up after baseline 20% or less?	NA	NA
14. Were key potential confounding variables measured and adjusted statistically for their impact on the relationship between exposure(s) and outcome(s)?	Yes	Yes
**Quality rating**	**Good**	**Good**

CD – Cannot determine, NA – Not applicable, NR – Not reported

Quality assessment of the included studies (for case-control studies) continued…

Criteria	Chen 2016	Smith 2016
1. Was the research question or objective in this paper clearly stated?	Yes	Yes
2. Was the study population clearly specified and defined?	Yes	Yes
3. Was a sample size justification, power description, or variance and effect estimates provided?	No	No
3. Was the participation rate of eligible persons at least 50%?	NR	NR
4. Were controls selected or recruited from the same or similar population that gave rise to the cases (including the same timeframe)?	Yes	Yes
5. Were the definitions, inclusion and exclusion criteria, algorithms or processes used to identify or select cases and controls valid, reliable, and implemented consistently across all study participants?	Yes	Yes
6. Were the cases clearly defined and differentiated from controls?	Yes	Yes
7. If less than 100 percent of eligible cases and/or controls were selected for the study, were the cases and/or controls randomly selected from those eligible?	NA	NA
8. Was there use of concurrent controls?	Yes	Yes
9. Were the investigators able to confirm that the exposure/risk occurred prior to the development of the condition or event that defined a participant as a case?	Yes	Yes
10. Were the measures of exposure/risk clearly defined, valid, reliable, and implemented consistently (including the same time period) across all study participants?	Yes	Yes
11. Were the assessors of exposure/risk blinded to the case or control status of participants?	No	No
12. Were key potential confounding variables measured and adjusted statistically in the analyses? If matching was used, did the investigators account for matching during study analysis?	Yes	Yes
**Quality rating**	**Good**	**Good**

CD – Cannot determine, NA – Not applicable, NR – Not reported
