# Supplementary material for: A systematic review and meta-analysis on the association between ambient air pollution and pulmonary tuberculosis
Source: Sci Rep. 2022 Jul 4;12:11282. doi: 10.1038/s41598-022-15443-9 (PMC9253106; doi:10.1038/s41598-022-15443-9)
Supplement: Supplementary file 7 — Supplementary Information 7. [file 41598_2022_15443_MOESM7_ESM.pdf]

Quality assessment of evidence on exposure to air pollutants and incidence of pulmonary tuberculosis

**Quality assessment of evidence on incidence of pulmonary tuberculosis**						**Summary of findings**	
						**Outcome/Total Participants**	
**Studies (Participants)***	**Limitation[1]**	**Inconsistency[2]**	**Indirectness[3]**	**Imprecision[4]**	**Publication bias[5]**	**Pooled Risk ratio (95% CI)**	**Overall Quality**
**$PM_{2.5}$**							
7 (63391)	Some limitations	Some inconsistency	No indirectness	No imprecision	Undetected	1.13 (1.06 – 1.20)	⊕⊕OO Low
**PM_{10}**							
10 (253788)	Some limitations	Some inconsistency	No indirectness	No imprecision	Undetected	1.05 (1.02 – 1.05)	⊕⊕OO Low
**CO**							
6 (63595)	Some limitations	Some inconsistency	No indirectness	Some imprecision	Undetected	1.01 (0.997 – 1.02)	⊕⊕OO Low
**NO_2**							
9 (128126)	Some limitations	Some inconsistency	No indirectness	No imprecision	Undetected	1.08 (1.03 – 1.13)	⊕⊕OO Low
**SO_2**							
10 (247988)	Some limitations	Some inconsistency	No indirectness	No imprecision	Undetected	1.06 (1.04 – 1.09)	⊕⊕OO Low
**O_3**							
6 (63595)	Some limitations	Some inconsistency	No indirectness	Some imprecision	Undetected	1.00 (0.99 – 1.02)	⊕⊕OO Low

(Participants)* - minimum number of participants as some studies did not report number of participants.

[1]Most studies reported limitations that could affect the generalizability of their findings.

[2]Despite similar measures of effect across the studies, there were significant heterogeneity and variability (I^2) across the studies.

[3]All patients and interventions studied were patients and interventions of interest.

[4]No imprecision except for CO and O_3 where there is some imprecision with the confidence intervals.

[5]No statistical evidence of publication bias
