# Supplementary material for: A systematic review and meta-analysis on the association between ambient air pollution and pulmonary tuberculosis
Source: Sci Rep. 2022 Jul 4;12:11282. doi: 10.1038/s41598-022-15443-9 (PMC9253106; doi:10.1038/s41598-022-15443-9)
Supplement: Supplementary file 8 — Supplementary Information 8. [file 41598_2022_15443_MOESM8_ESM.pdf]

The Collaboration for Environmental Evidence Synthesis Appraisal Tool (CEESAT)

Version 2.1 for Evidence Reviews (Last updated 4th March 2021)

The CEESAT checklist provides a point by point appraisal of the confidence that can be placed in the findings of an evidence review by assessing the rigour of the methods used by the review, the transparency with which those methods are reported and the limitations imposed on synthesis by the quantity and quality of available primary data.

Note that CEESAT does not distinguish between reviews that do not employ methodology that reduces susceptibility to bias and increases reliability of findings and reviews that may have employed such methodology but do not report it.

THE CEESAT CHECKLIST

Tick or fill one 'O' for each question. Start with the 'Gold' definition and move down the list if it does not apply. When 'AND' is used all statements must apply and when 'OR' is used one of the alternative should apply.

Please make a single decision for each question (or hypothesis). Many reviews address more than one question. This may be reflected in the review title being broad whilst subsections address more specifically defined questions. If this is the case please complete an assessment for each question and fill in multiple rows for the article on the assessment spreadsheet.

Please note that supplementary material to (and linked from) the article, including protocols, should be included in the assessment and are provided when noted by the Editorial Team. Please contact us if you have problems accessing supplementary material. Reference to methods of other original articles should not be included.

Please cite as: Collaboration for Environmental Evidence 2020. The Collaboration for Environmental Evidence Synthesis Appraisal Tool (CEESAT). Version 2.1.

1. THE REVIEW QUESTION

Rationale: A well-defined question (or hypothesis) is crucial for assessing the reliability of subsequent decisions on searching and screening for eligible studies, as well as forming the basis for critical appraisal of study conduct and for data extraction and synthesis.

1.1 Are the elements of the review question clear?

Tick whichever statement applies

- ☑ Gold: The review question or hypothesis is clearly stated and clearly defines key elements, (e.g. PICO, PECO, PO, PIT, etc.) correctly, such as the subject or population of interest, the intervention or exposure type, the comparator and valid measures of outcome.
- ○ Green: The review question or hypothesis is clearly stated, and key-elements are mentioned although not formally defined in terms of PICO, PECO, PO, PIT, etc.
- ○ Amber: The question or hypothesis is stated in broad terms but key-elements are unclear or poorly defined.
  OR
  Question or hypothesis not stated but problem or issue is stated such that a question can be inferred
- ○ Red: A question, hypothesis or problem is not stated
  OR
  There is no stated objective to provide an answer to a question or test of a hypothesis.
  OR
  The article does not contain an evidence synthesis (e.g. primary research or descriptive overview)

2. THE METHOD/PROTOCOL

Rationale: A protocol is a document describing the methods to be used, produced prior to the commencement of an evidence synthesis. It describes the background to the synthesis, the questions, the strategy that will be used to search for primary research articles, and the criteria for deciding whether or not an article is then relevant to include in the synthesis. The protocol should also outline the approach to assessing the quality of each included study, and to extracting and synthesising data from primary research studies. Writing a protocol is therefore analogous with developing and documenting a methodology prior to conducting fieldwork or experiments and is similarly integral to producing a study that is robust against post hoc changes in methods and scope.

2.1 Is there an a-priori method/protocol document?

- ☑ Gold: The review cites a separate a-priori protocol or documented pre-defined method containing details of proposed conduct of all review and synthesis stages (e.g. question, search, eligibility screening, critical appraisal, data extraction and synthesis)
AND
It is linked from the synthesis (e.g. as supplementary material or hosted on a separate website)
AND
It was publicly accessible prior to the conduct of the review
AND
It was submitted to an independent body for peer review and publication.
- ○ Green: The review cites a separate a-priori protocol or documented pre-defined method containing details of conduct of all review and synthesis stages (e.g. question, search, screening, critical appraisal, data extraction and synthesis)
AND
It is linked from the synthesis (e.g. as supplementary material or hosted on a separate website)
AND
It was publicly accessible prior to the conduct of the review.
- ○ Amber: The review cites a separate a-priori protocol or documented pre-defined method, but this does not contain all details of conduct of all review and synthesis stages or was not publicly accessible prior to the conduct of the review
OR
The review includes a defined methods section (not a-priori) listing the synthesis stages conducted and providing sufficient detail to enable the method to be replicated (therefore this standard is met only if all of criteria 3.1, 4.1, 6.1 & 7.1 are rated green or above).
- ○ Red: There is no protocol and the review methods are not clearly defined in the methods section of the review or there are no methods reported.

3. SEARCHING FOR STUDIES

An optimal search for literature should possess three key properties: comprehensive (maximises the number of potentially relevant studies found), systematic (avoiding ad hoc search strategies reduces the susceptibility to bias resulting from e.g. no defined endpoint of search) and transparent (readers should be able to replicate and evaluate the search).

3.1. Is the approach to searching clearly defined, systematic and transparent?

Rationale: Search strategies should be outlined in the predefined protocol or review methods. An optimal search for literature should aim to maximise comprehensiveness (aiming to identify all relevant studies) and transparency (readers should be able to replicate and evaluate the search). This is to avoid 'cherry-picking' studies or assembling a biased or unrepresentative body of evidence. Where possible, advice should be sought from an expert such as an information specialist/scientist.

- ☑ Gold: All search terms and search strings, with Boolean operators ('AND', 'OR' etc.) and wildcards, are clearly stated for each source (e.g. databases, search engines, specialist websites) so that the exact search is replicable by a third party
  AND
  There is information about the sources searched, together with dates of search and any limitations justified (e.g. languages, publication date, no grey literature searches).

- ○ Green: All search terms and search strings, with Boolean operators ('AND', 'OR' etc.) and wildcards, are clearly stated for each major source (e.g. databases, search engines) so that the exact search is replicable by a third party but search terms for minor sources (e.g. specialist websites), if used, may be missing.
  AND
  There is information about the sources searched and search options selected, together with dates of search but some limitations (reported or evident) not justified (e.g. languages or publication date or no grey literature searches)

- ○ Amber: The search is described but not adequately to be fully replicable by a third party either because the specific search terms are not stated or Boolean operators/wildcards are not stated (so it is unclear how the search terms are combined).
  OR
  There is information about the databases searched, but dates of search not given and no limitations justified (e.g. language or publication date or no grey literature searches).

- ○ Red: No information regarding the search strategy (search terms and strings) used.

3.2. Is the search comprehensive?

Rationale: The resources used to find relevant literature influence the comprehensiveness and reliability of the synthesis. The principal sources for locating peer-reviewed articles are electronic databases of scientific literature and academic search engines, with a range of supplementary methods. No single database indexes all peer-reviewed articles. Moreover, these sources are unlikely to capture potentially relevant grey literature (e.g., reports by governmental and non-governmental organisations, unpublished studies) and consequently can be complemented by searching thesis repositories, websites of relevant organisations and conducting internet searches. Other supplementary search strategies include citation chasing (backwards and forwards), and contact with experts in the field.

- ○ Gold: Sources of articles searched capture both conventionally published scientific literature and grey literature using a combination of databases, search engines and specialist websites (may also be informed by stakeholders) or limitations are fully justified.
  AND
  Comprehensiveness of search is tested using independent samples of articles (test lists should be provided) of the relevant literature to demonstrate adequate sensitivity.

NB. Statements such as *'We considered only peer-reviewed material because this is more reliable than grey literature'* without evidence that the methodological quality of potentially relevant grey literature was assessed do not indicate that grey literature was objectively considered.

- ✓ Green: Sources of articles searched are stated and capture both conventionally published scientific literature and grey literature using a combination of databases, search engines and specialist websites (may also be informed by stakeholders) or limitations are fully justified.

NB. Statements such as '*We considered only peer-reviewed material because this is more reliable than grey literature'* without evidence that the methodological quality of potentially relevant grey literature was assessed do not indicate that grey literature was objectively considered.

- ○ Amber: Resources used are stated but limited, without justification, to conventionally published scientific literature or just one or two sources.

- ○ Red: Resources used not stated or search is not systematic (i.e. studies appear to have been selected).

4. INCLUDING STUDIES

Comprehensive searches may generate a large number of articles that vary widely in their relevance to the synthesis. Authors must then determine whether or not each article is sufficiently relevant (eligible) for inclusion in the data synthesis stage. However, the choice of eligibility criteria can influence the conclusions of the synthesis, and the application of inadequately defined criteria can be subjective and lead to biases. Decisions over which studies are relevant for inclusion should therefore be based on clearly defined criteria, and should be replicable and transparent. Criteria 4.1-4.3 refer only to studies included/excluded on the basis of relevance to the review question – see 5 for inclusion/exclusion on the basis of methodological quality.

4.1: Are eligibility criteria clearly defined?

Rationale: Clearly stated criteria for eligibility decisions minimise the potential for subjective decisions to influence which studies are included in the review, increase the transparency of the synthesis, and allow readers to assess the validity of the criteria to the review question. In addition to following the review question, eligibility criteria may define limits on the type of primary research to be considered in terms of (for example): geographic scope, type of data reported, type of intervention or impact, study design, date.

- ☑ Gold: Eligibility criteria are precisely defined (e.g. reliance on broad and potentially ambiguous terms should be avoided) and expressly related to each key element of the question (other criteria such as study design may also be considered)
  AND
  Criteria are consistent between a-priori protocol and review or differences are fully explained.

- ○ Green: Eligibility criteria are precisely defined (e.g. reliance on broad and potentially ambiguous terms should be avoided) and are expressly related to each key element of the question (other criteria such as study design may also be considered).

- ○ Amber: The questions/scope/objectives of the review are stated such that the type of primary research articles/studies to be included are broadly apparent, but the review **does not** explicitly identify criteria expressly related to each key element of the question (other criteria such as study design may also be considered).
  OR
  Some eligibility criteria are defined but either incomplete or no clear relationship to a review question (possibly because the question is poorly defined).

○ Red: No to both amber criteria above (eligibility criteria are not stated).

4.2 Are eligibility criteria consistently applied to all potentially relevant articles and studies found during the search?

Rationale: More than one person should screen studies for inclusion to reduce the risk of human error and to ensure that the criteria are applied consistently to the articles returned by the search. If more than one person independently evaluates the relevance of the same articles, the consistency of inclusion/exclusion decisions can be assessed. Piloting the criteria, and discussing and refining the eligibility decisions can also ensure they are consistently applied.

☑ Gold: The eligibility criteria are independently applied by more than one reviewer to all of the screened articles/studies (at title screening stage, pragmatic decisions about dual screening of subsamples is justified e.g. because large numbers of titles were screened)
AND
Replicability of eligibility decisions was measured and reported and all disagreements between reviewers discussed so that the resolutions informed subsequent assessments.

○ Green: The eligibility criteria are independently applied by more than one reviewer to a sample of justified size of the screened articles/studies at title, abstract and full text.
AND
Replicability of eligibility decisions was measured and reported and all disagreements between reviewers discussed so that the resolutions informed subsequent assessments.

○ Amber**:** The eligibility criteria are applied by more than one reviewer to a sample of the screened articles/studies at abstract and full text but reviewer independence is uncertain (i.e. not reported) or absent.
AND
Replicability of eligibility decisions was examined (but a measure may not be reported) and all disagreements between reviewers discussed so that the resolutions informed subsequent assessments.

○ Red: Number of reviewers not reported
OR
Only one reviewer applied criteria at abstract or full text stage,
OR
Where two reviewers, consistency of decisions not tested/reported
OR
No eligibility criteria provided (see 4.1)

4.3 Are eligibility decisions transparently reported?

Rationale: Listing all articles that were screened for eligibility and indicating whether each was included or excluded in data synthesis (usually as supplementary material), makes it clear whether potentially relevant studies have been omitted according to the eligibility criteria or were not captured by the search. Documenting the reasons for article exclusion at full text is essential for transparency.

- ☑ Gold: The number of unique articles found during the searches (after removal of duplicates) is presented
  AND
  The number excluded at each stage of the screening process is fully presented (e.g. in a flow diagram or table)
  AND
  Reasons for exclusion of each article/study considered at full-text are presented (e.g. in an appendix)
  AND
  A list of eligible (included) articles/studies is presented as a separate list or in tables (not just included in reference list).

- ○ Green: The number of articles excluded at each stage of the screening process is reported but some aspects missing (e.g. number of unique articles or articles unobtainable)
  AND
  Reasons for exclusion of each article/study considered at full-text are presented (e.g. in an appendix)
  AND
  A list of eligible (included) articles/studies is presented as a separate list or in tables (not just included in reference list).

- ○ Amber: The number of articles excluded during the screening process is reported (or inferable) but some aspects missing (e.g. number of unique articles or articles unobtainable or reasons for exclusion at full text)
  AND
  A list of eligible (included) articles/studies is presented as a separate list or in tables (not just included in reference list).

- ○ Red: No to either or both of the amber criteria above

5. CRITICAL APPRAISAL

Primary research can vary widely in methodological validity (internal validity) and study context (external validity). Internal validity can influence the findings of the research, and, if not properly accounted for, the conclusions of syntheses that use it. External validity can influence the relevance/applicability of the study to users of the findings in individual contexts. Critical appraisal involves transparently evaluating the design and conduct of each included study based on methodologies, which can then help to objectively account for variation in study quality by placing greater emphasis on the most reliable studies.

5.1 Does the review critically appraise each study?

Rationale: Documented critical appraisal, as applied to each individual each included study, using relevant, pre-defined critical appraisal criteria allows the author(s) of the synthesis and the reader to make more objective assessments of the relative reliability (or weighting) of each study.

Some potentially relevant studies may not meet baseline methodological requirements (e.g. small sample size, pseudoreplication, spatial autocorrelation, lack of appropriate controls etc.) and so may be excluded from the synthesis. Effectively, these studies are weighted as 'zero'. Studies included in the synthesis may be treated differently according to the rigour of the sampling design, according to differences in sampling effectiveness (e.g. sample size, sampling area, study duration, etc.), or according to their generalisability for the synthesis in hand (e.g. spatial scale, study setting, etc.).

- ○ Gold: An effort is made to identify all sources of bias relevant to individual included studies (threats to internal and external validity)
  AND
  Each type of bias (threat to internal and external validity) is assessed and explained individually for all included studies
  AND
  Results are reported using an a-priori defined (in protocol) critical appraisal sheet.

NB. This does not include syntheses in which the design and conduct of each study are stated but validity is not explicitly considered (no critical appraisal), or in which methodological rigour is discussed without transparent and objective assessments for each study.

- ☑ Green: An effort is made to identify all sources of bias relevant to individual included studies (threats to internal and external validity)
  AND

Each type of bias or threat to internal and external validity is assessed individually for all included studies and reported on a critical appraisal sheet.

NB. This does not include syntheses in which the design and conduct of each study are stated but validity is not explicitly considered (no critical appraisal), or in which methodological rigour is discussed without transparent and objective assessments for each study.

- ○ Amber: Some characteristics of all included studies are explicitly identified as indicators of threats to internal and external validity of studies but not reported for individual studies.

- ○ Red: No critical appraisal conducted
  OR
  all critical appraisal criteria not applied to all individual included studies. This may include syntheses in which the methods for each study are stated but validity is not explicitly considered, or in which methodological rigour is discussed without transparent and objective assessments for each study.

5.2. During critical appraisal was an effort made to minimise subjectivity?

Rationale: Use of explicit appraisal criteria that are developed in advance of the process avoids post hoc decisions being made about study validity and changes to appraisal criteria during the assessment process. Employing more than one reviewer reduces susceptibility to bias in critical appraisal decisions or errors in judgement.

- ○ Gold: An effort is made to minimise subjectivity by predefining a critical appraisal process in a protocol
  AND
  At least two people independently critically appraised each study with disagreements and process of resolution reported.

- ☑ Green: An effort is made to minimise subjectivity by predefining critical appraisal process in a protocol
  AND
  At least two people critically appraised each study but not independently (e.g. second person aware of first person's decision)

- ○ Amber: At least two people critically appraised each study but not independently (e.g. second person aware of first person's decision) or subset of studies was appraised by at least two people independently. Disagreements and process of resolution MIGHT NOT be reported.

- ○ Red: No to Amber above (e.g. Only one person critically appraised each study or number not reported)
  OR
  No critical appraisal conducted (i.e. RED for 5.1 above).

6. DATA EXTRACTION

The volume and type of data collected by primary research articles varies substantially, even when similar questions are addressed. Authors of evidence syntheses must make decisions on which data to extract and on how to extract this information. These decisions may influence the findings of the synthesis, and so to minimise bias, the approach to data extraction should be clearly stated and, wherever possible, the extracted information should be comparable and consistent between studies.

6.1. Is the method of data extraction fully documented?

Rationale: Transparently identifying a consistent set of data to extract from each study, for example into a structured data extraction sheet, allows the process to be replicated and evaluated by a third party, and reduces the potential for bias over which data are extracted from individual studies. Typically, extracted information from each study included in the review comprises: study aims; intervention details, study design; population characteristics; comparator details and results (point estimates and measures of variance).

- ☑ Gold: The authors state in an a-priori protocol the type of data to be extracted
  AND
  the methods by which data from each study will be extracted so that the process can be replicated and confirm these methods were ultimately used in their report or reasons for deviation.
- ○ Green: The authors state in the methods (but not in an a-priori protocol) the type of data to be extracted
  AND
  the methods by which data from each study were extracted so that the process can be replicated (In some cases methods may have been partially reported in an a-priori protocol but then modified or substantially developed during the review process).
- ○ Amber: The authors state in the methods the type of data to be extracted
  AND
  although the review does not provide a fully replicable methodology for data extraction, it is possible to infer the broad method from the reported results (e.g. a table that lists all eligible studies and data extracted might be included).
- ○ Red: No to either part of amber above. It is not clear what data were selected for extraction and/or no consistent approach to data extraction is reported.

6.2. Are the extracted data reported for each study?

Rationale: Providing a summary in which the population, intervention/exposure and outcome for each study are stated makes data extraction transparent, and makes it easier for readers to locate the most relevant primary literature and conduct supplementary analyses if required (Liberati et al, 2009).

- ☑ Gold: All data selected for extraction are provided in a table or spreadsheet as set out in the a-priori protocol. This includes the data used in the synthesis from each primary study (e.g 'raw' outcome metrics: means, variance measures) and meta-data
  AND
  calculations or transformation of data by review authors using extracted data (e.g. effect sizes, averaging over variables summarisation of themes, coding, etc.) are reported in full and therefore replicable.
- ○ Green: All data selected for extraction are provided in a post-hoc table or spreadsheet. This includes the data used in the synthesis from each primary study (e.g 'raw' outcome metrics: means and variance measures) and meta-data
  AND
  calculations or transformation of data by review authors using extracted data (e.g. effect sizes, averaging over variables summarisation of themes, coding, etc.) are reported in full and therefore replicable.
- ○ Amber: The review provides a table/spreadsheet that includes some of the extracted metrics data for some or all studies (e.g. Table/spreadsheet only lists partial data for each study but omits other information OR Table/spreadsheet lists extracted data, but not for all studies OR A combination of these). Note: It may be unclear if all studies are included since they are not listed anywhere in the article.
- ○ Red: No to amber above. Data extracted are not presented.

6.3. Were extracted data cross checked by more than one reviewer?

Rationale: Checking data extraction improves accuracy by ensuring the correct data are extracted for each element and reduces the risk of errors due to interpretation or transcription

- ○ Gold: Data were extracted from each study by at least two independent reviewers.
- ☑ Green: An explanation was provided of how a sample of extracted data was cross checked between two or more reviewers.
- ○ Amber: A statement that cross-checking between two reviewers was carried out is provided but explanation unclear or incomplete.
- ○ Red: No report of cross checking is provided.

7. DATA SYNTHESIS

The approach to synthesising included studies varies substantially, and some approaches are more effective at ensuring objectivity and minimising potential bias than others.

7.1. Is the choice of synthesis approach appropriate?

Rationale: If appropriate, data should be pooled in a quantitative synthesis (e.g. meta-analysis, meta-regression). If substantial differences between populations, interventions, comparators or outcomes exist, meta-analysis (i.e. combining effect sizes across different studies) may not be appropriate. Since meta-analysis effectively treats all individual studies part of one large study, meta-analysis is only appropriate when calculating an average effect is meaningful. If it is not appropriate to pool data across studies in meta-analysis, a reason for this should be given, and structured approach to some other quantitative or narrative synthesis taken, with efforts made to make sense of the whole of the data set, beyond describing results from individual studies in turn, noting differences in the weight of evidence behind statements made, and appropriate use of table and graphical presentations of results. Vote-counting (summing the studies which gave positive or negative findings) is not an appropriate synthesis method as an indication of impact or effectiveness.

- ☑ Gold: The choice of synthesis method (i.e. quantitative or narrative synthesis) is pre-specified, described in sufficient detail to be replicable and justified (e.g. in the protocol) on the basis of scoping characteristics of included studies, taking into consideration variability between studies in sample size, study design, context, outcomes measures etc

  OR

  Justified post hoc as a deviation from the protocol (but still described in sufficient detail to be replicable) as a result of unexpected outcome (not pre-specified) of critical appraisal and data extraction

  AND

  Where quantitative and statistical approach to meta-analysis is not employed when it may have been appropriate, a justification for this should be given (e.g. studies too diverse or data not synthesisable).

- ○ Green: The choice of synthesis method (i.e. quantitative or narrative synthesis) is described in sufficient detail to be replicable and is (or appears) justified on the basis of characteristics of included studies, taking into consideration variability between studies in sample size, study design, context, outcomes etc.

  AND

Where quantitative and statistical approach to meta-analysis is not employed when it may have been appropriate, a justification for this should be given (e.g. studies too diverse or data not synthesisable).

- ○ Amber: No to either or both of the above (e.g. no justification for not undertaking meta-analysis when it may have been appropriate) but none of the listed criteria under Red below.
- ○ Red: No to either or both in green above:
  AND either
  quantitative synthesis (e.g. meta-analysis) is undertaken when inappropriate
  OR
  vote-counting relied on as an indicator of impact or effectiveness
  OR
  narrative synthesis does not include all studies or unclear if it includes all studies.

7.2 Is a statistical estimate of pooled effect (or similar) provided together with measure of variance and heterogeneity among studies?

Rationale: If a sufficient quantity and quality of data is available then the presentation and assessment of evidence can be much improved by providing statistical information. Some evidence reviews will be unable to do this because of limitations of the primary data.

- ○ Gold: Statistical estimates of findings are presented using pre-defined (e.g. in the protocol) meta-analysis method that justifies synthesis approach including study weighting and subgroup analysis
  AND
  Consideration is given to study independence (e.g. through sensitivity analysis) and bias (e.g. tests for publication bias).
- ✓ Green: Statistical estimates of findings are presented using meta-analysis method that justifies approach (e.g. using study weighting and subgroup analysis)
  AND
  Consideration is given to study independence (e.g. through sensitivity analysis) and bias (e.g. tests for publication bias).
- ○ Amber: Statistical estimates of findings presented using defined meta-analysis method but lacks justification of approach and/or consideration of study independence (e.g. through sensitivity analysis) and/or bias (e.g. tests for publication bias).
- ○ Red: No statistical estimate provided either because meta-analysis not conducted or not possible
  OR
  Statistical estimate provided but not clear what meta-analysis method was used.

7.3 Is variability in the study findings investigated and discussed?

Rationale: Studies can differ in their results (heterogeneity) which may be due to chance, but could also reflect variables other than the factor of interest that differ between studies (effect modifiers). The presence and magnitude of effect modifiers can reveal important information about a system. Investigating heterogeneity therefore indicates the degree to which effects are generalisable across taxa, regions etc., and is also necessary to evaluate the appropriateness of combining studies conducted on different populations or reporting different outcome metrics. These can be investigated statistically in meta-analysis through for example subgroup analyses, sensitivity analyses and meta-regression. In narrative syntheses differences in findings may be discussed in terms of differences in study design, context, population, focus etc.

- ○ Gold: A strategy for investigating effect modifiers is provided in an a-priori protocol and followed (or variations explained) in the review
  AND
  Effect modifiers (e.g. taxa being considered, location, habitat type, study design etc.) were investigated statistically through meta-analysis (alternatively, evidence for heterogeneity between studies is tested and reported as non significant)
  AND
  Authors have used results of critical appraisal (not just statistical weighting in meta-analysis) in their quantitative and/or narrative synthesis.
- ☑ Green: Effect modifiers (e.g. taxa being considered, location, habitat type, study design etc.) are investigated statistically through meta-analysis (alternatively, evidence for heterogeneity between studies is tested and reported as non significant)
  AND
  Authors have used results of critical appraisal in their quantitative and/or narrative synthesis.
- ○ Amber: Effect modifiers (e.g. taxa being considered, location, habitat type, study design etc.) investigated statistically through meta-analysis (alternatively, evidence for heterogeneity between studies is tested and reported as non-significant) but authors have not used results of critical appraisal in their quantitative and/or narrative synthesis.
  OR
  Effect modifiers (e.g. taxa being considered, location, habitat type, study design etc.) were investigated descriptively through narrative synthesis.
- ○ Red: Reasons for variability in study findings not investigated. Effect modifiers were not considered (this includes studies that use quantitative synthesis but fail to test for heterogeneity statistically).

8. LIMITATIONS

Rationale: All reviews will have limitations and it is important that authors are explicit about the known limitations of the primary data and the conduct of the review process. Here we acknowledge the subjective nature of this criterion and the appraiser must use some subjective judgement to decide on the adequacy of any statement on limitations.

8.1 Have the authors considered limitations of the synthesis?

- ○ Gold: An explicit section is devoted to the authors' consideration of limitations of their review including limitations of the primary data (available evidence), possible sources of bias in the review process, conduct of the review process and recommendations made for future syntheses and primary research.
- ☑ Green: An explicit section or identifiable passage of text is devoted to the authors' consideration of limitations of primary research/data and of conduct of the review process but does not consider all of the following: possible sources of bias in the review process, conduct of the review process and recommendations made for future syntheses and primary research.
- ○ Amber: Some consideration of limitations is evident but not explicitly stated or not focus of specific section
  OR
  Consideration of limitations of the primary research included but limitations of the conduct of the review not included.
- ○ Red: No evident consideration of limitations of primary data or review conduct.
